# ΔNp63α transcriptionally represses p53 target genes involved in the radiation-induced DNA damage response: ΔNp63α may cause genomic instability in epithelial stem cells

**DOI:** 10.1186/s13014-022-02139-7

**Published:** 2022-11-15

**Authors:** Ken-ichi Kudo, Naohiro Tsuyama, Kento Nagata, Tatsuhiko Imaoka, Daisuke Iizuka, Misaki Sugai-Takahashi, Moe Muramatsu, Akira Sakai

**Affiliations:** 1 Department of Radiation Life Sciences, Fukushima Medical University School of Medicine, Fukushima, Japan; 2 Department of Radiation Effects Research, National Institute of Radiological Sciences, National Institutes for Quantum Science and Technology, Chiba, Japan; 3 Department of Diagnostic Pathology, Fukushima Medical University School of Medicine, Fukushima, Japan

**Keywords:** ΔNp63α, TP63, p53, DNA damage response, Mammary epithelial cells, Stem cells, Genomic instability, Radioresistance

## Abstract

**Background:**

The DNA damage response (DDR) is a mechanism that protects cells against radiation-induced oxidative DNA damage by causing cell cycle arrest and apoptosis. *TP63* is a member of the tumour suppressor *TP53* gene family, and ΔNp63α, a *TP63* splicing variant, is constitutively expressed in the stem cell-containing basal layer of stratified epithelial tissues, including the mammary gland, where it plays a critical role in stemness and tissue development. ΔNp63α has been reported to transcriptionally inhibit the tumour suppression protein p53. This p53-repressive activity may cause genomic instability in epithelial stem cells exposed to radiation. In this study, we analysed the inhibitory effect of ΔNp63α on radiation-induced DDR.

**Methods:**

To elucidate the role of the p53-repressive effect of ΔNp63α in radiation response, we performed a p63-siRNA knockdown experiment using human mammary epithelial cells (HMECs) expressing ΔNp63α and then performed ectopic and entopic expression experiments using human induced pluripotent stem cells (hiPSCs). After irradiation, the expression of DDR-related genes and proteins in ΔNp63α-expressing and control cells was analysed by RT–qPCR, Western blotting, and flow cytometry.

**Results:**

The mRNA/protein expression levels of BAX and p21 were significantly increased in p63-siRNA-treated HMECs (sip63) after X-ray irradiation (4 Gy, 0.7 Gy/min) but not in scramble-siRNA treated HMECs (scr). Transcriptomic analysis showed decreased RNA expression of cell cycle-related genes and increased expression of programmed cell death-related genes in sip63 cells compared to scr cells. Furthermore, flow cytometric analysis revealed an increase in apoptotic cells and a decrease in 5-ethynyl-2´-deoxyuridine uptake in sip63 cells compared to scr cells. On the other hand, both the ectopic and entopic expression of ΔNp63α in apoptosis-sensitive hiPSCs reduced the expression levels of BAX after irradiation and significantly decreased the number of apoptotic cells induced by radiation.

**Conclusion:**

Taken together, these results indicate that ΔNp63α represses p53-related radiation-induced DDR, thereby potentially causing genomic instability in epithelial stem cells.

**Supplementary Information:**

The online version contains supplementary material available at 10.1186/s13014-022-02139-7.

## Background

Ionizing radiation is well known to induce oxidative DNA damage, such as DNA double-strand breaks (DSBs), and consequently trigger the DNA damage response (DDR), including cell cycle arrest and apoptosis. The DNA guardian protein p53 plays the most important role in DDR: it promotes DNA repair and the elimination of cells that are unable to repair the damage caused by oxidative stresses, including radiation. The p53 protein is normally degraded by the ubiquitin–proteasome system through the E3 ubiquitin ligase MDM2 and is maintained at low levels in a steady state [1]. However, when DSBs are induced in the nucleus by radiation, p53 is acetylated and phosphorylated by proteins such as p300/CBP and ataxia telangiectasia mutated (ATM), preventing its ubiquitination and consequent degradation and enabling its activation: it then binds to the promoter region of DDR-related genes, including *CDKN1A*, and upregulates their gene expression to promote DNA strand repair [2–4]. When DNA repair fails, the cell undergoes various forms of death including apoptosis. Notably, p53 induces apoptosis through the upregulation of pro-apoptotic genes, including *BAX*, and the subsequent activation of pro-apoptotic proteins, including Caspase-3 [5]. Thus, p53 is the key protein that directs the DDR.

*TP63* was discovered as a *TP53* family gene in 1998 and has high homology with *TP53* in its transactivation (TA), DNA-binding, and tetramerization domains [6, 7]. It has two main isoforms: TAp63 has an N-terminal TA homologous to that of p53, while ΔNp63 has a truncated but specific TA domain, the expression of which depends on selective promoters. In addition, these two isoforms each have three further isoforms, α, β, and γ, with different C-termini generated through alternative RNA splicing. In particular, the α-type of p63 (p63α) has an additional region in its C-terminus, which includes the sterile alpha motif (SAM) domain and plays an important role in protein–protein interactions [8, 9]. The TAp63 and ΔNp63 isoforms have opposing functionalities: TAp63 acts like p53, enhancing apoptosis and cell cycle arrest in the DDR, while ΔNp63 acts as a dominant negative regulator of TAp63; the α-type of ΔNp63 (ΔNp63α) is the most potent p53 repressor among all isoforms [6, 8, 10]. ΔNp63α is expressed only inside the nucleus, appearing as foci at replication factories upon immunostaining [11]. It has been elucidated that ΔNp63α cannot form hetero-oligomers with p53, unlike mutant p53 [12], and thus, its p53 repressor activity is attributed to competitive inhibition, which results from the high homology between its DNA-binding domain and that of p53, and gene regulations [10].

Yang et al. [6] and Westfall et al. [8] confirmed the transcriptional inhibitory effect of ΔNp63α on p53 through competitive binding to the p53 response element (RE) in the p21 promoter region by luciferase reporter assay and chromatin immunoprecipitation (ChIP). Interestingly, the magnitude of its inhibitory effect depends on the C-terminus; among all isoforms ΔNp63α has the greatest inhibitory effect on p53, while ΔNp63β and ΔNp63γ have only a weak effect. In addition, some studies have shown an inverse relationship between ΔNp63α expression and apoptosis [13, 14]. On the other hand, Woodstock et al. [10] recently noted that transcriptome analyses show very little overlap in the target sequences of ΔNp63α and p53 [15]; this observation presents a problem, as it is inconsistent with the competitive inhibition theory. Min et al. [14] reported that ΔNp63α promotes the expression of the follistatin (Fst) gene, which inhibits apoptosis by blocking the binding of Activin to its receptor located on the cell membrane. Thus, the inhibitory effect of ΔNp63α may involve two pathways: competitive inhibition and antagonistic gene expression.

ΔNp63α is highly expressed in the basal cell layer of the epithelium and plays an important role in stemness maintenance and cell repopulation [6, 16]. The basal cells in epithelial tissues contain stem cells and have relatively high reproductive ability and stemness [17–19]. These cells differentiate into other cell types as ΔNp63α expression decreases [20]. On the other hand, stem cells are considered favoured candidates for transformation into cancerous cells because of their inherent capacity for self-renewal and their longevity, which may allow the accumulation of genetic mutations induced by oxidative stresses over long periods [21, 22]. Radiation-induced carcinogenesis has also been found to occur with higher frequency in the epithelium, including the mammary gland and epidermis [23]. Recently, its immunostaining has also been regarded as a marker for the diagnosis of squamous cell carcinoma [24, 25]. Thus, ΔNp63α is thought to confer stem cell properties, while its p53 repressor activity is expected to be an important factor for elucidating the mechanism of radiation-induced carcinogenesis.

Radiation is one of the best stimulants with which to study DDR, as it leads to the oxidation and breakage of DNA strands through the generation of reactive oxygen species (ROS) in the vicinity of the DNA. Thus, radiation biology is one of the most appropriate fields for elucidating the DDR-related function of ΔNp63α. However, few studies have focused on the transcriptional repressor activity of ΔNp63α in this field. Two studies have previously reported that mammary basal cells are less responsive to radiation than mammary luminal cells, differentiating cells from basal stem/progenitor cells [26, 27]. If basal stem cells that have genetic mutations and chromosomal aberrations continue to divide and differentiate for a long time, cancerous cells may be expected to develop. In this study, we aim to clarify transcriptional inhibition mediated by ΔNp63α during the radiation response.

## Methods

### Materials

To analyse the function of ΔNp63α, human mammary epithelial cells (HMECs, Thermo Fisher Scientific, Waltham, MA, USA) were purchased and cultured in HuMEC Ready Medium (1X) (HuMEC, Thermo Fisher Scientific) supplemented with gentamicin/amphotericin solution (Thermo Fisher Scientific). DeltaNp63alpha-FLAG (#26,979; RRID:Addgene_26979) was obtained from Addgene (www.addgene.org), and pRetro-X-Tight-Pur and pRetro-X-Tet-Off Advanced vectors were purchased from TaKaRa Bio Inc. (Otsu, Japan). Polymerase chain reaction (PCR) primers were obtained from Merck (St. Louis, MO, USA) (Table S1). Knockdown experiments were carried out by lipofection with siRNA targeting the DNA-binding domain of *TP63* mRNA with siLentFect (Bio-Rad, Hercules, CA, USA). Scramble siRNA (scr) was used as a negative control. All siRNA double strands were synthesized by NIPPON GENE (Tokyo, Japan) (Table S1). G418 sulfate (Nacalai Tesque, Kyoto, Japan) and puromycin (TaKaRa Bio Inc.) were used for cell selection. Doxycycline (Dox, TaKaRa Bio Inc.) was used to regulate gene expression using an inducible Tet-OFF system.

### Irradiation

Cells in microtubes, plates, and dishes were irradiated with X-rays using an MBR-1605R X-ray generator (HITACHI, Hitachi, Japan) at 150 kVp and 5 mA with a 0.5 mm Al + 0.2 mm Cu filter. The dose rate was 0.7 Gy/min.

### iPSC cell culture, transfection, and virus packaging

Human induced pluripotent stem cells (hiPSCs, HiPS-RIKEN-2 A; RRID:CVCL_B512), which were derived from human fibroblasts and have high radiosensitivity, were obtained from the Riken Cell Bank (Tsukuba, Japan). hiPSCs were seeded on a plate coated with iMatrix-511 (Nippi, Tokyo, Japan) and cultured with StemFit AK02N (REPROCELL, Yokohama, Japan) containing 10 µM Y27632. hiPSC-ΔNp63α (iPS-DN) with Dox-dependent ΔNp63α expression was generated by retrovirus infection as described below. DeltaNp63alpha-FLAG plasmids were digested with BamHI and NotI, and the fragment containing the ΔNp63α coding sequence was separated by agarose gel electrophoresis and purified with a FastGene Gel/PCR Extraction Kit (NIPPON Genetics, Tokyo, Japan). The ΔNp63α fragment was ligated downstream of the pRetro-X-Tight-Pur vector (puromycin-resistant), which has a tight TRE promoter, with the Ligation high reagent (TOYOBO, Osaka, Japan). For retrovirus packaging, plasmids were transfected into gp293 cells (RRID:CVCL_E072) using PEImax 40,000 (Polyscience Inc., Warrington, PA, USA) following the manufacturer’s protocol. Virus particles were recovered by centrifugation of culture supernatant supplemented with PEG6000 and NaCl [28]. Virus particles were also produced from the pRetro-X-Tet-Off Advanced vector (Neomycin-resistant, TaKaRa Bio Inc.). Then, hiPSCs were coinfected with these virus suspensions in the presence of polybrene (4 µg/mL) and cultured with medium containing 1 µg/mL puromycin, 200 µg/mL G418 sulfate, and 10 ng/mL Dox. After incubation for 14 days, Dox was removed from the medium, and ΔNp63α expression was confirmed by both reverse transcription-quantitative PCR (RT–qPCR) and Western blotting. Subsequently, 5 passages were performed to obtain cells for use in experiments.

### Organoid culture and immunohistochemistry (IHC)

Human mammary organoids were generated as described previously [27]. HMECs were mixed on ice with HuMECs containing 2 mg/mL rat tail collagen I (Corning Inc., Corning, NY, USA). Four hundred microlitres of this mixture was plated in the wells of an ultralow-attachment 24-well plate (Corning Inc.) and then incubated at 37 °C and 5% CO_2_ to allow collagen gelation. After 1 h, the gels were immersed in HuMECs supplemented with 2.5% FBS, 10 µM forskolin (Enzo Biochem Inc., Farmingdale, NY, USA), and 0.5 µg/mL hydrocortisone (STEMCELL Technologies, Vancouver, Canada). The medium was changed every 4 days for 11–14 days. Thereafter, mammary organoids embedded in gels were fixed in 4% paraformaldehyde (PFA) for 15 min at room temperature (RT) and processed for regular paraffin embedding. Immunofluorescence and haematoxylin–eosin (HE) staining for mammary organoids were performed on paraffin-embedded sections. Antigen retrieval was accomplished by autoclaving at 120 °C in 10 mM sodium citrate buffer (pH 6.0) for 15 min. After blocking with 5% normal goat serum (Vector Laboratories, Burlingame, CA, USA), sections were treated with primary antibodies overnight, followed by secondary antibodies at RT for 1 h (Table S2). Finally, sections were mounted with Vectashield mounting medium containing 4´,6-diamidino-2-phenylindole (DAPI, Vector Laboratories). Images were captured using an Axio Imager. Z2 (RRID:SCR_018856; Carl Zeiss, Oberkochen, Germany).

### Immunocytochemistry (ICC)

Cells were inoculated on a sterile coverslip in dishes and incubated at 37 °C and 5% CO_2_. After X-irradiation, the cells were fixed with 4% PFA for 15 min, permeabilized with 0.2% Triton X-100 in PBS for 30 min, and incubated with 5% normal goat serum to block nonspecific binding to the target sites in the cells. Cells were then incubated with primary antibodies at RT overnight, followed by incubation with secondary antibodies for 1 h at RT. Cells were mounted with Vectashield containing DAPI. Images were captured using an Axio Imager Z2. The primary and secondary antibodies used in this study are listed in Table S2.

### Neutral comet assay

To quantify the DSBs induced by radiation, neutral comet assays were performed. The cells were mixed with 0.5% low-melting-point agarose (Nacalai Tesque) and then seeded on microscope slides coated with 0.8% normal-melting-point agarose gel (Nacalai Tesque). After the agarose gel was solidified on ice for 15 min, the slides were immersed in alkaline lysis buffer (1% Triton X-100, 10% DMSO, 100 mM EDTA, 2.5 M NaCl, 10 mM Tris-HCl, 1% sodium lauryl sarcosine, pH = 10) for 1 h at 4 °C to release DNA from the cell and remove proteins. Then, slides were electrophoresed (13–15 V, 70–100 mA) in TAE buffer at 1 h and fluorescently stained with SYBR Gold Nucleic Acid Gel Stain (Thermo Fisher Scientific) for 30 min. To estimate the quantity of DSBs generated in a single cell, the %tail DNA was calculated using Comet Assay Software Project ver. 1.2.3b2 (RRID:SCR_007249; CaspLab).

### Flow cytometry (FCM)

For the measurement of DNA synthesis ability and cell cycle analysis, 5-ethynyl-2´-deoxyuridine (EdU) incorporated in cellular DNA was stained by using a Click-iT Plus EdU Flow Cytometry Assay kit (Thermo Fisher Scientific). The cells were treated with medium containing 10 µM EdU for 30 min before cell recovery, and EdU was incorporated into S-phase cells during DNA synthesis. After centrifugation at 300 x g for 5 min at 4 °C, the cells were washed with HBSS, fixed in 4% PFA and permeabilized with saponin-based solution according to the manufacturer’s protocol. Then, the RNA in the cells was degraded by 100 µg/mL RNase A. Finally, the cells were stained with 3 µM propidium iodide (PI, Thermo Fisher Scientific) for 15 min at RT.

For apoptosis analysis, FCM measurement by anti-Cleaved Caspase3 (CC3) antibody (RRID:AB_2341188; Cell Signaling Technology, Danvers, MA, USA) was performed on the irradiated cells. After fixation using 4% PFA and permeabilization with 0.1% Triton X-100, the cells were treated with CC3 antibody for 2 h on ice, followed by secondary antibody (Table S2).

To determine the ROS induced in X-irradiated cells, 2´,7´-dichlorodihydrofluorescein diacetate (DCFH-DA, Dojindo Laboratories, Kumamoto, Japan) was used as a colorimetric cell-permeable probe. HMECs were seeded on 60 mm dishes with a cell density of 1 × 10^6^ cells/dish, and DCFH-DA dye solution (λ_ex_ = 505 nm, λ_em_ = 525 nm) was added for 30 min before X-irradiation. Then, the cells were trypsinized, detached from the dish and analysed by FCM.

All FCM analyses were performed using an S3e cell sorter (RRID:SCR_019710; Bio-Rad). Data were analysed with FlowJo software version 10.8 (RRID:SCR_008520; BD Biosciences, Franklin Lakes, NJ, USA).

### RT–qPCR

Total RNA was extracted from cells by using Sepasol-RNA I Super G (Nacalai Tesque) and then reverse-transcribed into complementary DNA using SuperScript IV VILO Master Mix reverse transcriptase (Thermo Fisher Scientific) according to the manufacturer’s protocol. RT–qPCR was performed using SYBR Premix Ex Taq (TaKaRa Bio Inc.) and the LightCycler Nano (Roche Diagnostics, Basel, Switzerland). Primer sequences are listed in Table S1. The cycling profile included a hot start at 95 °C for 120 s; 40 cycles consisting of a denaturation step at 95 °C for 10 s, annealing at 60 °C for 20 s, and extension at 72 °C for 10 s; and fluorescent signal acquisition at 72 °C, with a final dissociation curve analysis. All gene expression levels were normalized to glyceraldehyde-3-phosphate dehydrogenase (GAPDH) as an internal control. The relative mRNA expression level of each gene was defined based on the threshold cycle (Ct) and calculated by the 2^−ΔCt^ formula.

#### RNA-seq

Total RNA was extracted from HMECs at 24 h post-irradiation by using a FastGene RNA Premium Kit (NIPPON Genetics). To ensure the accuracy of the data, each sample was mixed with total RNA obtained from two independent experiments. RNA quality was checked using agarose electrophoresis (total RNA > 1.0 µg, OD_260/280_ = 1.8–2.2, RIN > 6.5). RNA-seq was performed via next-generation sequencing using DNBSEQ-G400RS (RRID:SCR_017980; MGI Tech Co., Ltd., Shenzhen, China). All analyses were performed using integrated Differential Expression and Pathway analysis (iDEP) version 0.95 (http://bioinformatics.sdstate.edu/idep95/) [29].

### Western blotting

The cells were lysed in RIPA buffer with a protease inhibitor cocktail (Nacalai Tesque). After 30 min on ice, the cell lysates were centrifuged at 12,000 x g for 20 min at 4 °C, and the supernatants were recovered. The protein content of every sample was determined by the Bradford method, and equal amounts of protein from each sample were separated by SDS–PAGE and then transferred onto a nitrocellulose membrane (Bio-Rad) or a PVDF membrane (ATTO Corporation, Tokyo, Japan). The membrane was incubated with primary antibody and horseradish peroxidase-labelled secondary antibody. Then, the signal was visualized with Chemi-Lumi One Super (Nacalai Tesque) and detected with a ChemDox XRS+ (Bio-Rad). All antibodies used in this experiment are listed in Table S2.

### ChIP–qPCR

ChIP–qPCR was carried out using a SimpleChIP Enzymatic Chromatin IP Kit (Cell Signaling Technology) according to the manufacturer’s protocol. Briefly, iPS-DNs (4 × 10^6^ cells/sample) were treated with 2% PFA for 15 min at RT. The chromatin was harvested and fragmented using enzymatic digestion. Chromatin solutions were subjected to immunoprecipitation with anti-p53 (clone 7F5) antibody (RRID:AB_10695803; Cell Signaling Technology) overnight at 4 °C. Then, the immunoprecipitated complex was treated with protease. ChIP DNA was subjected to qPCR assay with amplification of the *BAX* and *CDKN1A* promoters using the primers listed in Table S1. Rabbit immunoglobulin G (IgG) was used as a negative control for nonspecific immunoprecipitation of DNA. The data were analysed by the following formula: %Recovery = 100 × 2^(input Cq − Target sequence Cq)^.

### Differentiation of hiPSCs into keratinocytes

Human iPSC-derived keratinocytes (iPS-KCs) were generated as described previously [30]. Briefly, hiPSCs were seeded and cultured under feeder-free conditions as described above, and then the culture medium was replaced with defined keratinocyte serum-free medium (DKSFM, Thermo Fisher Scientific) supplemented with 1 µM retinoic acid (Sigma–Aldrich) and 10 ng/mL human bone morphogenetic protein 4 (Peprotech, Cranbury, NJ, USA). Three days later, the culture medium was replaced with DKSFM supplemented with 20 ng/mL epidermal growth factor (FUJIFILM, Tokyo, Japan) and 10 µM Y27632. After an incubation for 7–14 days, the cells were reseeded on dishes coated with iMatrix-511.

## Results

### ΔNp63α inhibits the radiation-induced DDR through transcriptional repression

**Fig. 1:**
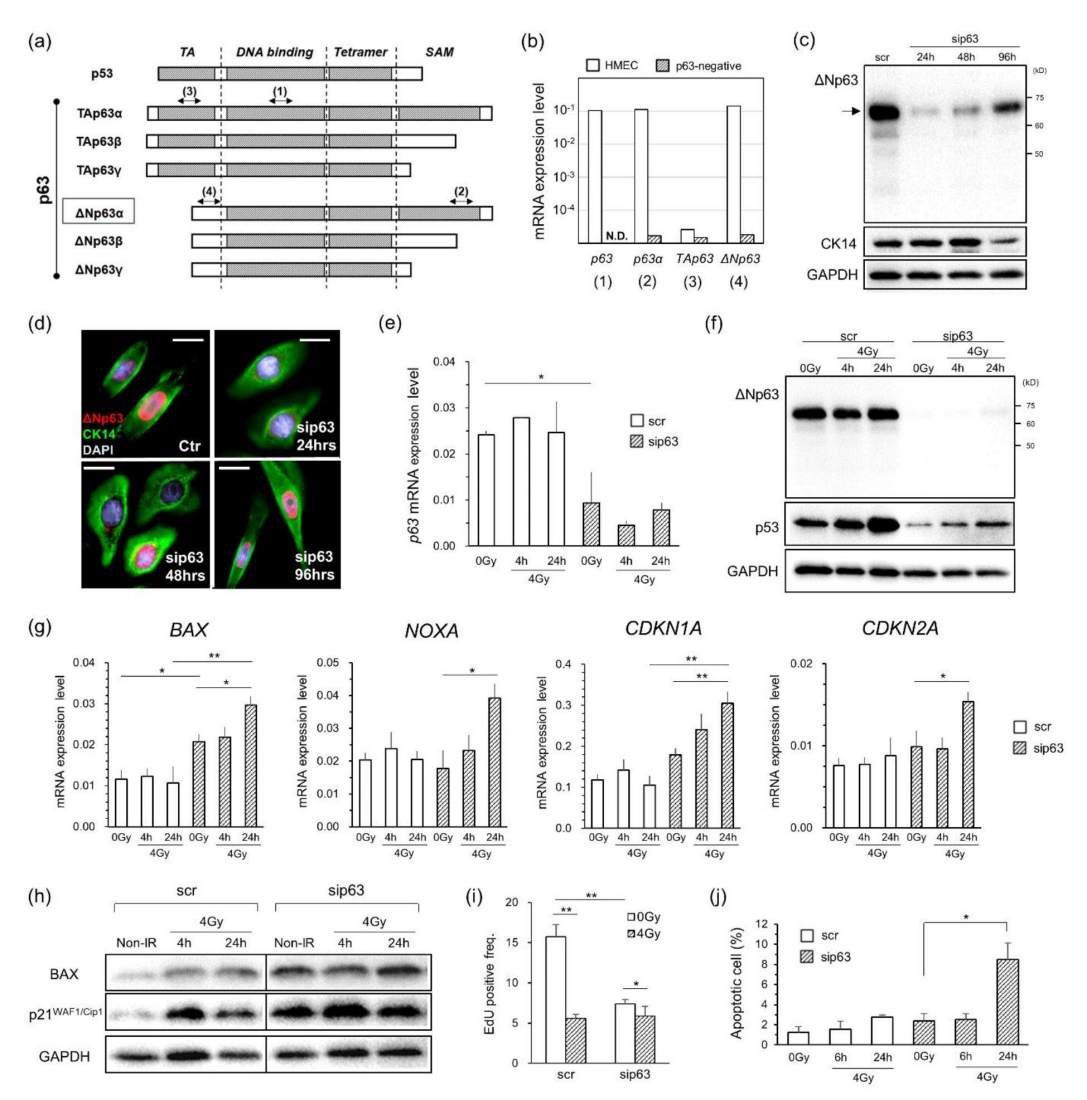
ΔNp63α knockdown experiments with HMECs. (a) Domain structure of human p53 and p63 isotypes. TAp63 and ΔNp63, highlighting the transactivation (TA), DNA-binding, oligomerization, and SAM domains. (b) mRNA expression concordant with positions (1)-(4) shown in Fig. 1a. hiPSCs were used as a negative control for p63. (c) Time-dependent variation in ΔNp63α and cytokeratin 14 (CK14) protein expression in p63 siRNA (sip63)- or scramble siRNA (scr)-treated HMECs. The arrow indicates the ΔNp63α protein band. (d) Representative immunofluorescence (IF) images of HMECs treated with sip63 and stained with ΔNp63 and CK14 antibodies. Red and green indicate ΔNp63 and CK14, respectively. Scale bar, 10 μm. (e, f) Time-dependent variations in *ΔNp63* mRNA (e) and ΔNp63α protein (f) contained in whole-cell extracts of HMECs treated with sip63 for 24 h after irradiation. (g) Measurement of DNA damage response (DDR)-marker mRNA expression in sip63-treated HMECs. (h) Western blotting analyses of BAX and p21 proteins. (i) Comparison of EdU-positive frequencies in sip63- and scr-treated cells, which was evaluated by flow cytometry (FCM). (j) Frequencies of apoptotic cells detected by FCM in sip63- or scr-treated HMECs. All values in mRNA expression data were scaled to the expression level of *GAPDH* as an internal control. Data represent the means and SEs of at least three independent assays. *P < 0.05, **P < 0.01 by Student’s *t* test

**Fig. 2:**
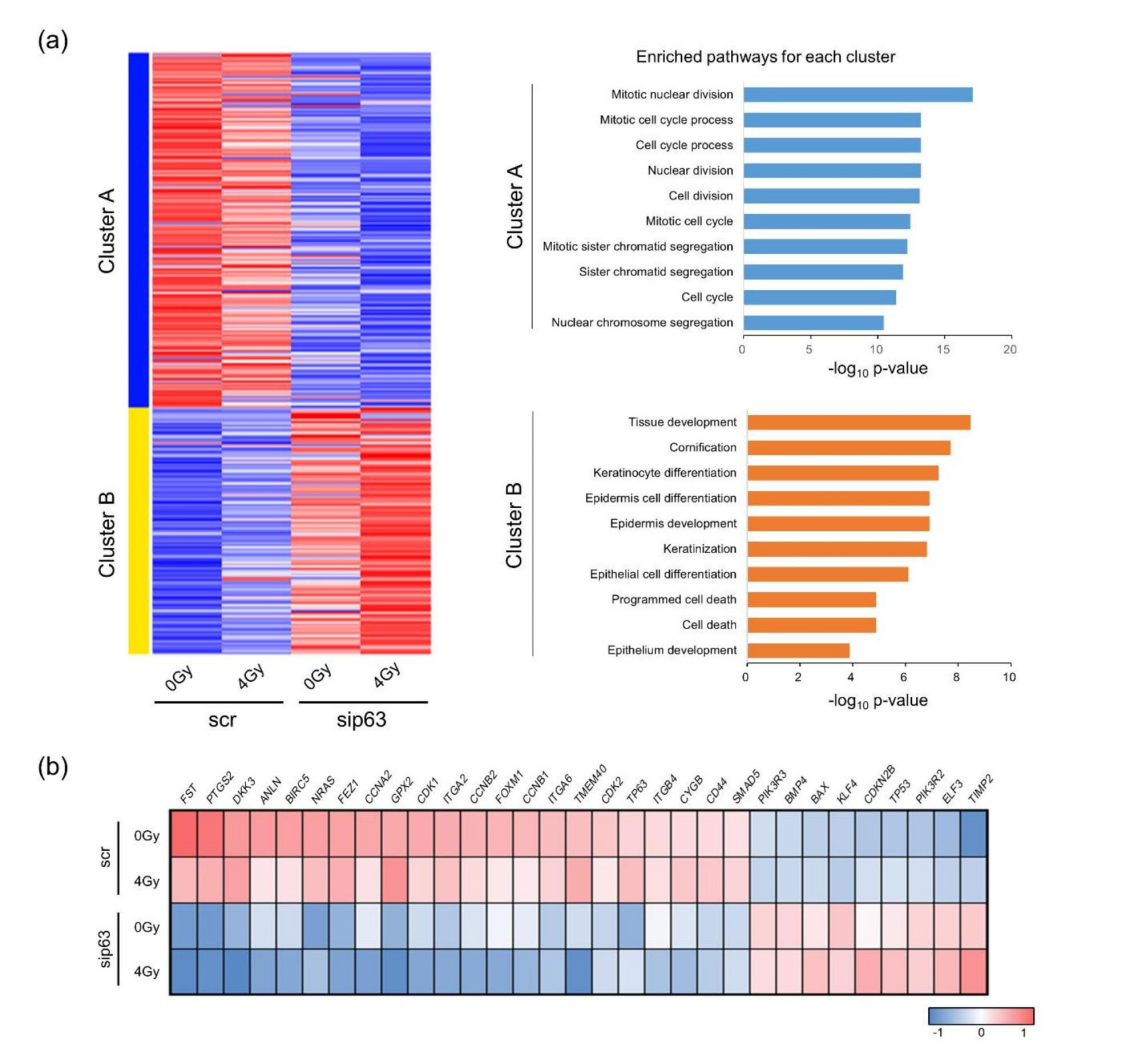
RNA transcriptome analysis of HMECs. (a) Left: K-means clustering heatmaps showing gene expression in siRNA-treated HMECs at 24 h post-irradiation. Right: Pathway enrichment analysis of scr- and sip63-treated groups in each cluster based on the Gene Ontology (GO) biological process database. Clusters A and B contain 295 and 205 genes, where upregulated and downregulated genes are depicted in red and blue, respectively. (b) Biclustering analysis based on the BCCC method for siRNA-treated HMECs at 24 h post-irradiation

To investigate the function of ΔNp63α as a p53 repressor in the radiation response, we first performed siRNA knockdown experiments in HMECs expressing the mammary basal cell markers cytokeratin (CK) 5/14 and integrin α6 (CD49f). HMECs were confirmed by FCM analysis to be a single population and expressed ΔNp63α (72 kDa) almost exclusively among all p63 isotypes in the nucleus (Fig. 1a-d and Fig. S1a and b). After p63-siRNA (sip63) treatment, ΔNp63α mRNA and protein were reduced to 10–20% of the levels in scr-treated cells at 24 h, but at 96 h, they had recovered to the level observed before sip63 treatment (Fig. 1c and d and Fig. S1c). At this time, the expression of genes upregulated by ΔNp63α, such as CK14 [31] and Fst [14], was decreased (Figs. 1c and 2b and Fig. S1e).

The sip63 sequence used in this study targets the DNA-binding domain of p63 (NM_001114980.2: position 714–732) and is not homologous to any other gene, as confirmed by NCBI BLAST (https://blast.ncbi.nlm.nih.gov/Blast.cgi) and GGGenome (https://gggenome.dbcls.jp/). RNA-seq and RT–qPCR results showed that *TP53* mRNA expression was increased after sip63 treatment (Fig. 2b and Figs. S1d and S3). Since the objective in this study was to examine the p53 repressor activity and functionality of ΔNp63α in radiation-induced DDR, we chose to irradiate the cells between 30 and 42 h after sip63 treatment when ΔNp63α was sufficiently attenuated and p53 expression was constant (Fig. S1d). The ΔNp63α expression level remained almost unchanged within 24 h after irradiation (Fig. 1e and f), while the p53 expression level showed time-dependent enhancement (Fig. 1f). On the other hand, the amount of p53 detected in the scr-treated group was more abundant than that in the sip63-treated group. In the study of radiation-induced breast cancer, experimental animals, such as rats, are exposed to 4 Gy of γ (X) irradiation to induce tumour formation [32]. Therefore, the dose used in this experiment was set at 4 Gy.

Radiation-induced DDR in sip63-treated cells was investigated using RT–qPCR and RNA-seq. RT–qPCR quantified the expression levels of the apoptosis-related genes *BAX* and *NOXA* and the cell cycle arrest-related genes *CDKN1A* and *CDKN2A*, which are regulatory targets of the p53 protein (Fig. 1g). After irradiation, p53 is phosphorylated by kinases such as ATM to escape ubiquitin degradation and binds to the cis-elements of these genes to activate transcription [2, 3]. In the scr-treated group, the expression level of each gene increased slightly within 24 h after irradiation (no significant difference). In contrast, in the sip63-treated group, these gene expression levels showed a significant time-dependent increase (Fig. 1g). In particular, the responses of *BAX* and *CDKN1A* were remarkable, and there was a significant difference in *BAX* between the scr-treated and sip63-treated groups even in the absence of irradiation (Fig. 1g, P_0Gy − 0 Gy_<0.05). To confirm this result, we examined the temporal changes in BAX and p21 after irradiation by Western blot analysis (Fig. 1h). The results showed that the overall expression of both proteins was higher in the sip63-treated group, which was consistent with the results of RT–qPCR analysis.

To further confirm these results, we measured the EdU uptake rate and radiation-induced apoptotic cells by FCM analysis (Fig. 1i and j). After sip63 treatment, the EdU uptake rate showed a decrease from approximately 15–7%, while the percentage of cells in G_0_/G_1_ phase increased to approximately 90% (Fig. 1i and Fig. S2a). After irradiation to the sip63-treated group, the EdU uptake rate showed a significant decrease from 7.4 to 5.9% (P < 0.05, Fig. 1i). The detection of apoptotic CC3-positive cells after irradiation showed that the percentage of apoptotic cells increased in a time-dependent manner within 24 h post-irradiation, ranging from 2% to approximately 10% (Fig. 1j and Fig. S2b). These results are consistent with the results of mRNA and protein analyses (Fig. 1g and h). In the scr-treated group, the proportion in S phase was also decreased at 24 h post-irradiation, and the G_0_/G_1_ phase population became dominant (Fig. S2a). The p21 protein is a strong CDK inhibitor and inhibits the G_1_-S phase transition [2, 3]. Therefore, this result is consistent with the results of the *CDKN1A* mRNA and p21 protein analyses (Fig. 1g and h). The cell viability in the sip63-treated group at 48 h post-irradiation was 67%, which was approximately 10% lower than that in the scr-treated group (Fig. S1f). This result may also indicate an increase in radiation-induced apoptotic cells in the sip63-treated group.

To validate the results of the siRNA-based knockdown experiments, we also performed ΔNp63α knockdown experiments using the CRL4^CRBN^-thalidomide system (Fig. S1g). Thalidomide is a celebron (CRBN) modulator that has been reported to bind to the E3 ubiquitin ligase substrate receptor, thereby enhancing the binding of CRBN to the neosubstrate ΔNp63α and contributing to the ubiquitination of ΔNp63α [33]. Thalidomide treatment of HMECs for 24 h resulted in 60% knockdown of ΔNp63α at the protein level (Fig. S1g, left panel). The cell viability measured by trypan blue staining was more than 90% for both 10 µM and 100 µM thalidomide. In HMECs irradiated at 24 h after thalidomide treatment, *CDKN1A* mRNA expression increased in a time-dependent manner (P < 0.05) (Fig. S1g, right panel).

### ΔNp63α regulates the cell proliferation and apoptosis pathways

We performed nonhierarchical clustering analysis using the k-means method on 500 genes that showed significant variation in RNA-seq data and classified them into two clusters (Fig. 2a). In Cluster A, the expression of each gene was highest in the nonirradiated scr-treated group and lowest in the post-irradiation sip63-treated group, and genes related to cell division were enriched in this cluster (Fig. 2a). This result is consistent with the results described above, where p63 knockdown decreased the expression of genes involved in cell proliferation, and the radiation response further arrested the cell cycle, indicating that ΔNp63α is strongly involved in cell proliferation. On the other hand, Cluster B was enriched in pathways related to cell differentiation, tissue development, cell death, and apoptosis, and the expression of each gene was lowest in the nonirradiated scr-treated group and highest in the post-irradiation sip63-treated group, in contrast to Cluster A (Fig. 2a). These results of enriched pathway analysis are consistent with previous studies reporting that ΔNp63α upregulates genes involved in cell cycle progression, stemness, and stem cell maintenance while downregulating genes involved in apoptosis and cell differentiation [11, 14, 15, 34]. Furthermore, biclustering analysis showed that the expression of genes involved in the cell cycle and stem cell maintenance, such as *CDK1/2* and *ITGA6/ITGB4*, was decreased in the sip63-treated group, while the expression of apoptosis-related genes and tumour suppressor genes, including *BAX*, *TP53*, and *PTEN*, was increased (Fig. 2b and Fig. S3). After irradiation, the expression of CDK inhibitors, such as *CDKN1A*, and p53-related apoptotic genes, such as *BAX* and *NOXA*, was increased (Fig. 2b and Fig. S3). On the other hand, the expression of apoptosis resistance genes, such as *Fst* and *BIRC5*, was higher in the scr-treated group than in the sip63-treated group (Fig. 2b and Fig. S1e).

### Analysis of the radiation-induced DDR in mammary organoids

**Fig. 3:**
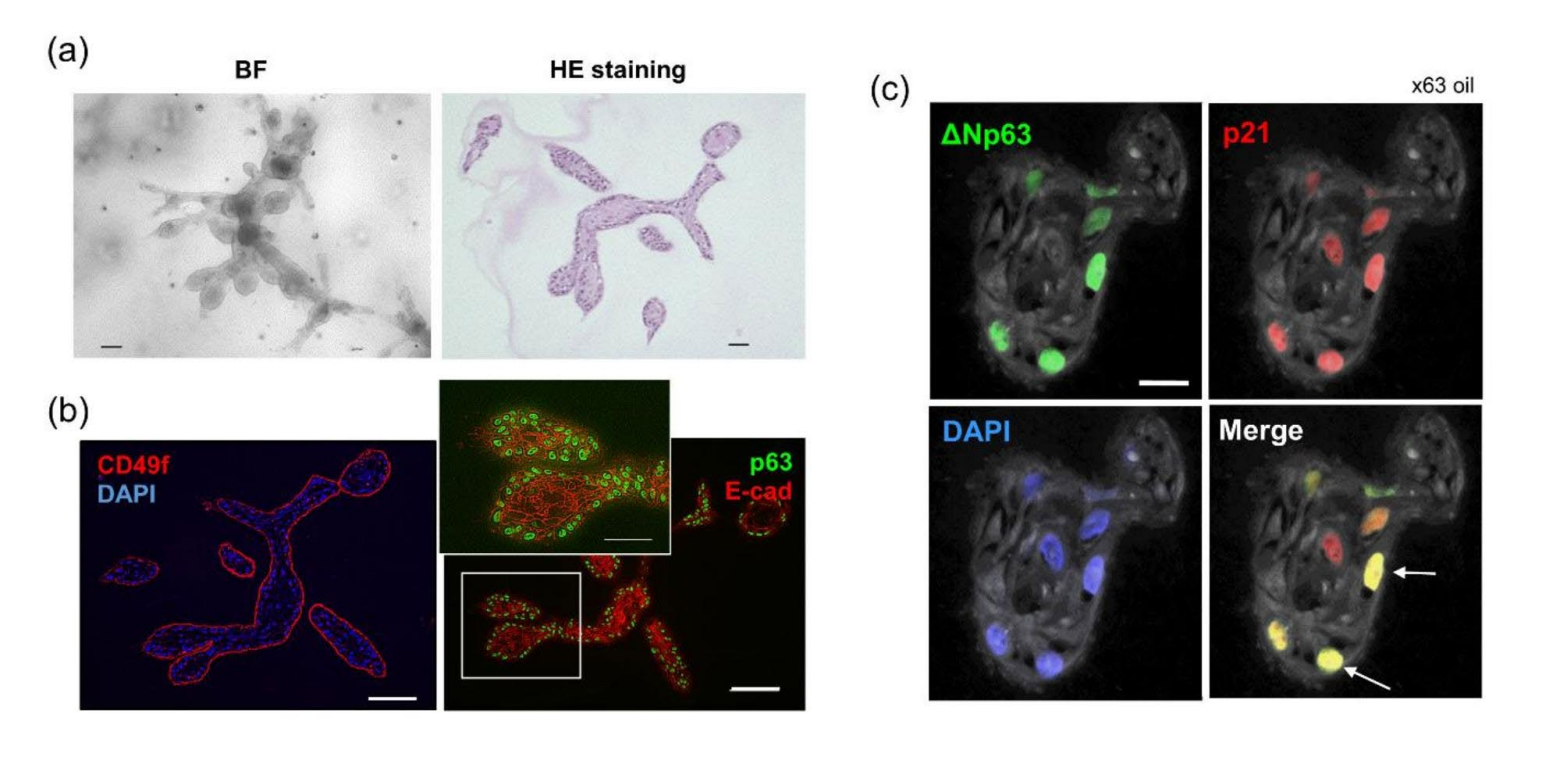
Radiation-induced DDR of mammary organoids. (a) Whole-mount bright field (BF) and (b) IF imaging of CD49f and p63 (4A4) in the mammary organoids. Scale bars are 50 μm for BF images and 100 μm for IF images. (c) Representative immunohistochemistry images of alveolar tissue in mammary organoids. Red, green, and blue indicate ΔNp63, p21, and DAPI, respectively. Scale bar, 20 μm

In siRNA knockdown experiments, we observed the radiation response of HMECs as described above, but the duration of p63 knockdown was too short, and the experiments were conducted under conditions where the expression of ΔNp63α and the genes that it regulates fluctuated dynamically, so the overall role of ΔNp63α in the radiation response could not be fully dissected. It has been thought that mammary stem cells are present among the basal cells because regenerative mammary glands are produced when mammary basal cells isolated from primary mammary cells by cell sorting are transplanted into rodent mammary fat pads [17, 18]. In addition, it has been recently reported that 3D culture of human and rat mammary basal cells in collagen gels yields mammary organoids that resemble in vivo structures [27, 35]. Therefore, we developed mammary organoids with both ΔNp63α-expressing and non-ΔNp63α-expressing cells by culturing collagen-embedded HMECs and observed the expression of p21 by immunostaining after irradiation. First, single HMECs formed two main types of structures: spherical colonies and mammary organoids (Fig. 3a, b and Fig. S4b). Mammary organoids typically showed structures with a diameter of 1 mm that have branches and acini, similar to the terminal ductal lobular units of mammary epithelium (Fig. 3a). The frequency of mammary organoid formation was approximately 1%. The cells outlining the organoid were positive for ΔNp63α and the basal marker CD49f (Fig. 3b). In general, basal cells of epithelial tissues such as mammary, prostate, and salivary glands strongly express ΔNp63α, and when ΔNp63α is attenuated, they differentiate and show the properties of luminal cells [20]. The organoids produced from a single HMEC in this study consisted of ΔNp63α-positive cells on the outer side and ΔNp63α-negative cells on the inner side, which is consistent with the findings of Centonze et al. [20]. When these organoids were irradiated, the expression of p21 was generally positive in both ΔNp63α-positive and ΔNp63α-negative cells of the acinus region (Fig. 3c and Fig. S4a).

### Protective role of ΔNp63α against radiation-induced DNA damage

**Fig. 4:**
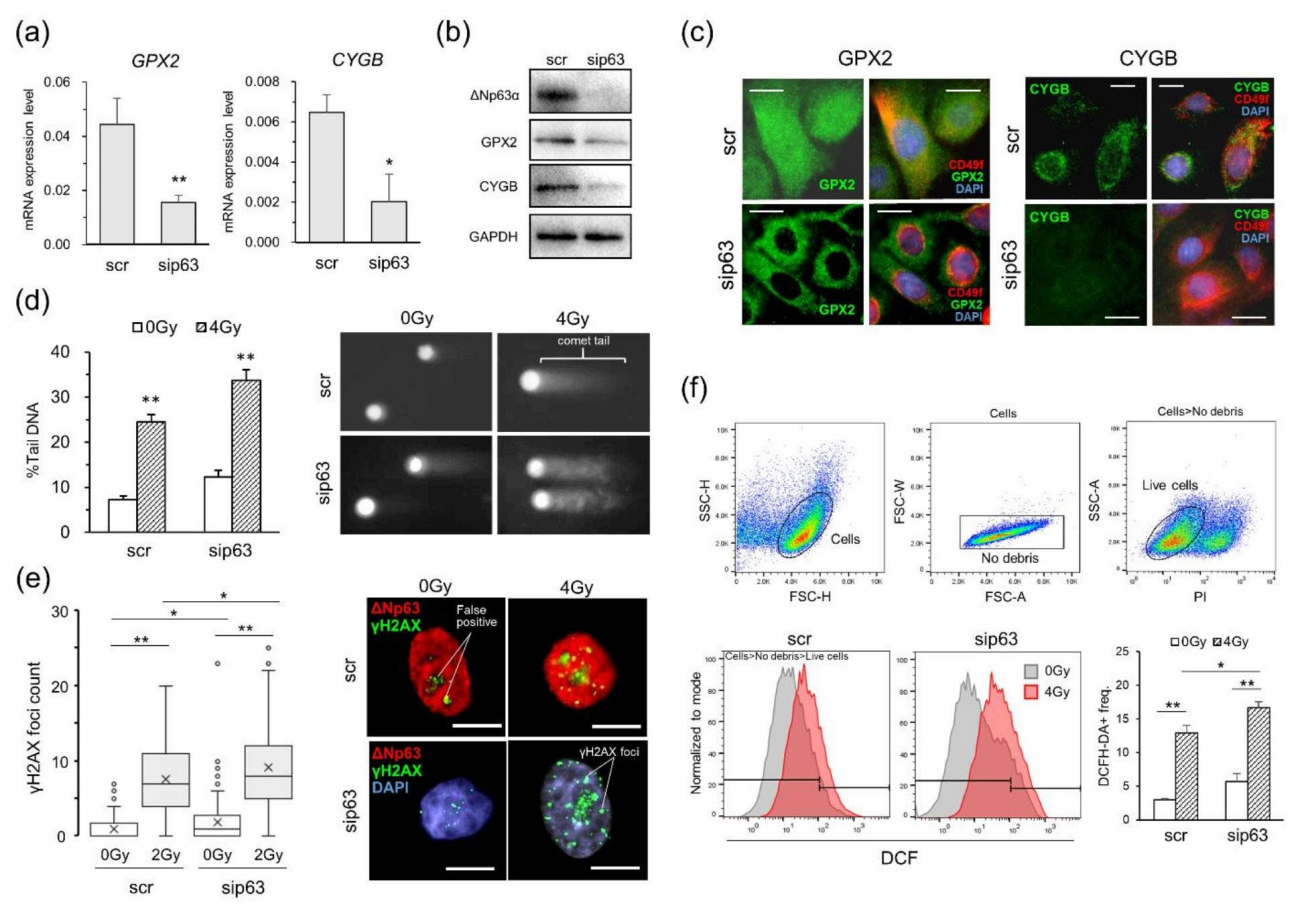
Inhibitory effects of GPX2 and CYGB expression on DNA lesions caused by radiation in HMECs. (a) *GPX2* and *CYGB* mRNA expression levels measured by RT–qPCR. Data are the means and SEs of at least three independent assays. (b) Detection of GPX2 and CYGB protein expression in HMECs by Western blotting analysis. (c) The localization of GPX2 and CYGB proteins inside cells. CD49f was used as a HMEC marker. Scale bar, 10 μm. (d) Determination of double-strand breaks (DSBs) using a neutral comet assay. The left panel shows the %tail DNA calculated from the comet tails shown in the right panel (n = 100). (e) Measurement of γH2AX foci observed in the nucleus at 4 h post-irradiation (n = 50). The left panel shows the numbers of γH2AX foci per cell. The right panel shows IF images of γH2AX foci, where green, red, and blue indicate γH2AX, ΔNp63, and DAPI, respectively. In the bottom images, DAPI was used as a counterstaining dye instead of ΔNp63. (f) FCM detection of intercellular reactive oxygen species (ROS) generated in HMECs immediately after X-irradiation. DCFH-DA, one of the major DCF derivatives, and PI were used as probe dyes for detecting ROS and dead cells, respectively. Data are the means and SEs of at least three independent assays. *P < 0.05, **P < 0.01 by Student’s *t* test

Glutathione peroxidase-2 (GPX2) and cytoglobin (CYGB), which are upregulated by ΔNp63α, have been reported to reduce ROS in cells and thereby protect cells [36–38]. In this study, sip63 treatment decreased the expression of GPX2 and CYGB (Figs. 2b and 4a-c), raising a possibility that the sip63 treatment might drastically increase radiation-induced damage itself via decrease of these enzymes with antioxidative functions. The previous experiment showed that ΔNp63α knockdown increased cell responsiveness to radiation, but since the expression of genes with antioxidant effects was also reduced by ΔNp63α knockdown, this effect could also be due to increased DNA oxidative damage, such as DSBs. Therefore, we directly quantified radiation-induced DSBs and intracellular ROS before and after ΔNp63α knockdown. To quantify the radiation-induced DSBs, we performed a neutral comet assay on the siRNA-treated group after irradiation to directly quantify DSBs [39, 40]. The experimental results showed that %tail DNA in both siRNA-treated groups was increased significantly after irradiation (Fig. 4d, P_0Gy − 4Gy_<0.01). Regardless of irradiation, approximately 5% more %tail DNA was detected in the sip63-treated group than in the scr-treated group, but there was no significant difference between the treatment group. To confirm this result, we further estimated the amount of DSBs from γH2AX foci counts detected by immunostaining. When chromosomal DNA is subjected to radiation to induce DSBs, the H2AX S139 sites around DSBs are phosphorylated by kinases such as ATM; H2AX becomes γH2AX [41, 42]. Therefore, by counting these foci, we can determine the number of DSBs produced by radiation. We counted the γH2AX foci induced in the siRNA-treated groups after radiation, and the number of DSBs generated was compared. After irradiation, γH2AX foci showed a significant increase in both treatment groups (Fig. 4e, P_0Gy − 2Gy_<0.01). In addition, the sip63-treated group showed a slight increase compared with the scr-treated group (Fig. 4e, P_0Gy − 0Gy, 2Gy−2Gy_<0.05). To further determine whether ΔNp63α expression affects intracellular ROS, we quantified radiation-induced intracellular ROS using DCFH-DA, which permeates cell membranes and is deacetylated by esterases localized in the cytoplasm, allowing it to react with ROS such as hydrogen peroxide (H_2_O_2_) and hydroxyl radicals, resulting in conversion to fluorescent 2’-7’ dichlorofluorescein (DCF) [43]. The experimental results showed that intracellular ROS was increased significantly in both irradiated treatment groups (Fig. 4f, P_0Gy − 2 Gy_<0.01). Similar to the results described above, intracellular ROS levels in the sip63-treated group were increased by 3% compared to those in the scr-treated group (Fig. 4f, P_4Gy − 4 Gy_<0.05, P_0Gy − 0 Gy_=0.07). These experimental results showed that the amounts of DSB and ROS were slightly increased in the sip63-treated group compared to the scr-treated group, indicating that ΔNp63α plays a role in protecting the genomic DNA from oxidative damage by upregulating antioxidant proteins, such as GPX2 and CYGB, and eliminating the intracellular ROS generated by cellular activities. However, it is less potent against radiation that directly generates ROS near DNA in the cell nucleus, indicating that ΔNp63α-mediated antioxidant regulation is not directly involved in controlling the amount of DNA damage caused by radiation.

### Verification of the DDR inhibitory effect of ΔNp63α by ectopic and entopic expression

**Fig. 5:**
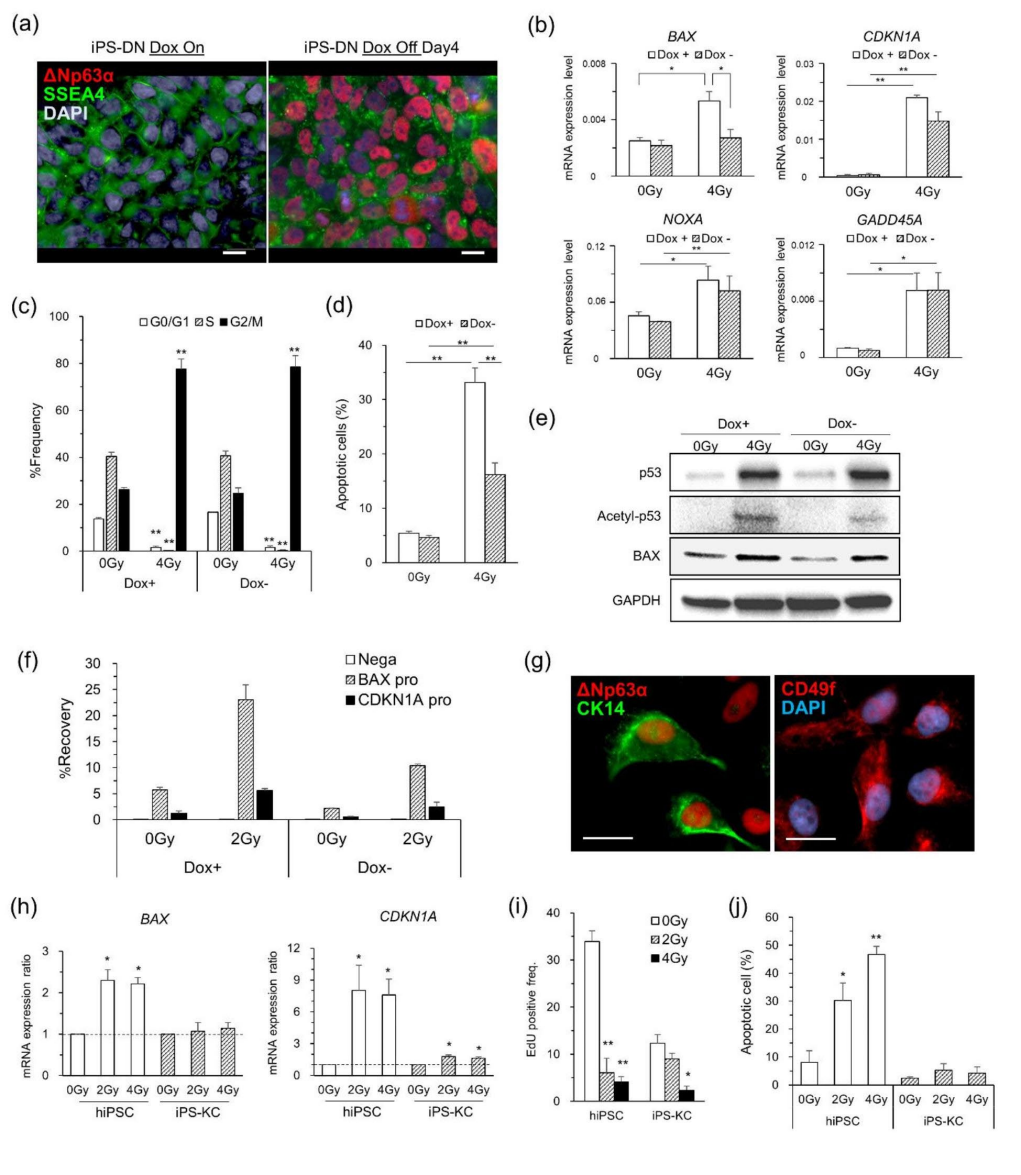
Analyses of iPS-DNs and iPS-KCs expressing ΔNp63α ectopically and entopically, respectively. (a) Immunostaining images of ΔNp63α in iPS-DNs ectopically expressing ΔNp63α with the doxorubicin (Dox) Tet-off system. Scale bar, 20 μm. (b) DDR marker mRNA expression in iPS-DNs post-irradiation. All values were scaled to the expression level of *GAPDH* as an internal control. Data represent the means and SEs of three independent assays. (c) The frequencies of cell cycle phase in iPS-DN with or without ΔNp63α expression at 24 h after X-irradiation, which were detected by FCM. (d) CC3-positive apoptotic cells detected by FCM in iPS-DNs post-irradiation. (e) Western blotting analysis for iPS-DNs post-irradiation. (f) ChIP analysis of *BAX* and *CDKN1A* promoters by anti-p53 antibody in iPS-DNs. Data represent the means of triplicate experiments. Nega: negative control, pro: promoter. (g) Immunostaining images for iPS-KCs entopically expressing ΔNp63α. Scale bar, 20 μm. (h) mRNA expression ratio of *Bax* and *CDKN1A* in iPS-KCs post-irradiation (*P < 0.05 vs. 0 Gy by Mann–Whitney U test). (i) Measurement of EdU-positivity frequency in iPS-KCs. (j) Apoptotic cell frequencies in hiPSCs and iPS-KCs at 24 h after irradiation. Data in all figure panels except (h) were analysed with Student’s *t* test (*P < 0.05, **P < 0.01)

ΔNp63α knockdown experiments using HMECs and analysis of organoids revealed that ΔNp63α expression suppresses radiation-induced DDR by inhibiting the transcription of p53-related genes. To verify this, we next observed whether ectopically or entopically expressed ΔNp63α exerted a similar inhibitory effect on the radiation-induced DDR. In this experiment, we used hiPSCs, which are highly susceptible to radiation-induced apoptosis, and generated two types of iPSCs: hiPSCs ectopically expressing ΔNp63α under the Tet-off control (iPS-DN) and hiPSC-derived keratinocytes entopically expressing ΔNp63α (iPS-KC). ΔNp63α expression, which was introduced into hiPSCs by a retroviral vector, was confirmed 3–5 days after Dox removal (Fig. 5a and Fig. S5a). The expression levels of four DDR-related genes, *BAX*, *CDKN1A*, *NOXA*, and *GADD45A*, in the Dox^+^ iPS-DNs 24 h post-irradiation were significantly increased compared to those in the nonirradiated cells, while the expression of *BAX* in the Dox^−^ iPS-DNs was not increased (Fig. 5b). Consistent with this result, protein analysis by Western blotting showed that BAX protein after irradiation was attenuated in Dox^−^ iPS-DNs compared to Dox^+^ iPS-DNs (Fig. 5e). We also performed ChIP–qPCR using an anti-p53 antibody and examined the change in p53 binding to target sequences before and after ΔNp63α expression. The results showed that p53 in Dox^+^ iPS-DNs bound directly to both the *BAX* and *CDKN1A* promoters post-irradiation, while p53 in Dox^−^ iPS-DNs repressed binding to these promoters (Fig. 5f). FCM analysis showed that the number of apoptotic cells was significantly decreased in Dox^−^ iPS-DNs compared to Dox^+^ iPS-DNs, which corroborated the results above (Fig. 5d and Fig. S5e). In addition, similar knock-in experiments using human B-cell-derived iPS cells (BiPSC-DN) [44] showed that BAX protein expression after irradiation was attenuated in Dox^−^ BiPSC-DN cells compared to Dox^+^ BiPSC-DN cells (Fig. S5f-h). On the other hand, cell cycle analysis showed almost the same response in Dox^+^ iPS-DN and Dox^−^ iPS-DN (Fig. 5c). The measurement of mRNA expression by RT–PCR showed that the increase in *CDKN1A* expression after irradiation was smaller in Dox^−^ iPS-DN cells than in Dox^+^ iPS-DN cells, although the difference was not significant (Fig. 5b). The mRNA expression level of *GADD45A*, which is also transactivated by p53 and involved in G2 phase arrest, was almost the same in Dox^+^ iPS-DN and Dox^−^ iPS-DN (Fig. 5b). This result was supported by the cell cycle analysis by FCM, which showed that each iPS-DN group underwent cell cycle arrest at G2 or M phase after irradiation (Fig. 5c and Fig. S5d).

In ectopic expression experiments, ΔNp63α suppressed gene transcription due to p53-activated DDRs, especially for *BAX*. We further confirmed this result by entopic expression experiments. The differentiation of hiPSCs into human keratinocytes is an established technique used in previous studies [30]. The iPS-KCs differentiated from hiPSCs in this study showed proliferation in a cobblestone-like fashion and expressed hallmarks, including ΔNp63α, CD49f, and CK14, similar to primary human keratinocytes (Fig. 5g and Figs. S5a and S6a, d and e). The CD49f/CD71 ratio, an index of stem cell enrichment in keratinocytes, was comparable between iPS-KCs cultured with collagen I + fibronectin, which has been used as a coating material in previous studies, and those cultured with iMatrix-511, used in this study (Fig. S6a, right panel). After irradiation, *BAX* and *CDKN1A* mRNA expression levels were significantly increased in hiPSCs, while in iPS-KCs, *CDKN1A* showed a significant increase, but *BAX* showed only a slight increase (Fig. 5h). The frequency of EdU uptake significantly decreased after both 2 and 4 Gy irradiation in hiPSCs but only after 4 Gy irradiation in iPS-KCs (Fig. 5i and Fig. S6b). The number of apoptotic cells detected by FCM showed a significant increase in hiPSCs but not in iPS-KCs (Fig. 5j and Fig. S6c). These FCM results were consistent with those of RNA expression analysis.

## Discussion

In this study, we experimentally dissected p53 repression by ΔNp63α during the radiation response. *TP63* is a member of the *TP53* gene family and is homologous to *TP53* in its TA, DNA-binding domain, and tetramerization regions. ΔNp63 is one of the proteins generated through selective alternative splicing after *TP63* transcription. Therefore, since its discovery, ΔNp63, especially ΔNp63α, has been thought to work competitively with p53 against target genes, thereby inhibiting the typical function of p53. On the other hand, radiation is highly cell permeant and induces DNA oxidative damage, including DSBs, which causes gene mutations and chromosomal aberrations through the generation of ROS, such as hydroxyl radicals and singlet oxygen, in the vicinity of DNA in the nucleus. This in turn leads to the activation of kinases such as ATM to induce the p53-derived DDR, including cell cycle arrest and apoptosis. Therefore, radiation biology provides the environment necessary to determine how ΔNp63α affects DDR in these signalling transductions and whether it is a competitive inhibitor of p53.

We first performed siRNA knockdown experiments using HMECs, which exhibit mammary basal cell characteristics, and observed changes in the expression of DDR-related genes downstream of p53 by RT–qPCR. After the X-irradiation to HMECs, genes related to cell cycle arrest and apoptosis showed little response in the scr-treated group but exhibited a marked increase in expression after ΔNp63α knockdown. This was further confirmed by Western blotting and RNA-seq. Although these DDR-related genes are upregulated through p53 binding to the promoter regions after irradiation, the differential expression of these genes even in the absence of irradiation suggests that ΔNp63α suppresses the expression of these genes at all times.

To investigate whether the transcriptional repression of DDR-related genes is independent of the amount of DNA damage, we directly quantified the DSBs generated in HMECs after irradiation. We found that the amounts of DSBs and ROS generated after irradiation were increased in the sip63-treated group compared to the scr-treated group. However, similar increases were observed in the nonirradiated group, suggesting that the difference between groups was due to the upregulation of antioxidant genes, such as *GPX2* and *CYBG*, by ΔNp63α [36, 37], resulting in a decrease in long-lived ROS, such as H_2_O_2_. Hence, ΔNp63α-expressing cells may be resistant to less reactive long-lived ROS. However, the ΔNp63α-mediated cellular antioxidant system is not sufficiently potent to protect DNA from more reactive short-lived ROS, such as hydroxyl radicals, generated in the vicinity of DNA by radiation, which thus did not explain the impact of ΔNp63α in radiation-induced DDR.

The RNA-seq analysis of HMECs revealed that ΔNp63α upregulates genes related to the cell cycle and cell division while downregulating genes related to apoptosis and cell death. These findings are consistent with those of previous studies [13, 14] and suggest that ΔNp63α acts as a transcription factor that maintains the stemness/inhibits the differentiation of mammary stem cells, keeps them alive by preventing cell death, and turns on genes that promote proliferation. The properties of ΔNp63α are opposite those of cancer suppressor genes such as *TP53* and *PTEN*, and it is thus thought that ΔNp63α initially inhibits the typical radiation responses triggered upon radiation-induced DNA damage. In addition, RNA-seq analysis suggests that ΔNp63α downregulates *TP53* and *PTEN*. These findings suggest that ΔNp63α suppresses the expression of *TP53* and other tumour suppressor genes and counteracts their effects. On the other hand, the protein analysis of HMECs showed that the p53 protein was upregulated by ΔNp63α expression, which was not observed in iPS-DNs. This suggest that ΔNp63α may increase the lifetime of the p53 protein in HMECs. Indeed, since the SAM domain in the C-terminus of ΔNp63α interacts with p300/CBP [9], it is possible that p53 undergoes acetylation, resulting in low expression but a longer lifetime. Taken together, the results indicate that the relationship between ΔNp63α and tumour suppressor genes requires further investigation.

In the three cell types used in this study, HMECs, iPS-DNs, and iPS-KCs, RT–qPCR analysis results showed that ΔNp63α significantly inhibited the expression of *BAX*. Consistent with this, the detection of apoptotic cells by FCM showed that ΔNp63α suppressed radiation-induced apoptosis, since the proportion of apoptotic cells decreased in ΔNp63α-expressing cells. Furthermore, ChIP–qPCR assays of iPS-DNs confirmed that the binding of p53 to its target gene promoter region was reduced by ΔNp63α expression. These results all suggest that ΔNp63α inhibits radiation-induced apoptosis by suppressing the expression of apoptosis-related genes such as *BAX* by p53. On the other hand, although ΔNp63α transcriptionally repressed *CDKN1A* expression and reduced p21 at the protein level, cell cycle analysis by FCM showed that cell cycle arrest occurred after irradiation regardless of ΔNp63α expression. A potential explanation for this result is that G1 arrest is regulated by genes or pathways independent of p53, such as ATM-CHK2-CDC25A [45]. Consistent with this model, the analysis of mammary organoids also showed that p21 is expressed at the same level in ΔNp63α-positive cells as in ΔNp63α-negative cells after irradiation. With regard to cell cycle arrest, this study supports the results of Westfall et al. [8]: ΔNp63α transcriptionally represses *CDKN1A*, but p21 is still expressed in certain amounts after transcriptional repression; moreover, the results of the cell cycle analysis by FCM suggest that the transcriptional repressive effect is limited during cell cycle arrest. At the same time, *GADD45A* in X-irradiated iPS-DNs was not transcriptionally inhibited, although it is a p53 downstream gene that has been shown to play a minor role in G2 arrest compared to ATM/CHK2 and ATR/CHK1 [46]. Thus, these results indicate that the transcriptional repression of ΔNp63α preferably affects proapoptotic p53 target genes. Understanding whether this transcriptionally repressive effect is due to competitive inhibition against p53 RE, as conventionally suggested, or to gene expression regulated by ΔNp63α will require further discussion addressing a wider range of p53-related genes.

It is problematic that stem cells contained in basal cells may all have this DDR vulnerability. If epithelial stem cells expressing ΔNp63α are vulnerable to DDR, especially apoptosis, it can be inferred that not only the mammary gland but also the prostate, lung, skin, and other epithelial tissues are at risk. Since caspase-3, which is activated by the p53 pathway, is also inhibited in ΔNp63α-expressing cells, it is conceivable that stem cells, which are long-lived and capable of differentiating into other cells [21, 22], may survive the failure to repair radiation-induced DNA damage, leaving DNA damage and mutations behind, and then differentiate into other cells, including cancerous cells. BRCA1/2 genes are involved in DNA repair, especially homologous recombination repair, which precisely repairs DSBs. Women with BRCA1 or 2 gene deficiency are more likely to develop breast cancer. Thus, this may suggest an additive or synergistic effect of BRCA gene deficiency and p53 repression by ΔNp63α. Indeed, BRCA1-deficient breast cancers are positive for CK14, which seems to be associated with the development of triple-negative breast cancer [47].

Epithelial stem cells such as mammary stem cells still lack definitive markers, and therefore, single-cell analysis, including single-cell RNA-seq and whole-genome sequencing, will be needed to elucidate DDR inhibition by ΔNp63α in epithelial stem cells and then characterize the mutational signatures and chromosomal aberrations induced by irradiation. The process of long-term mutation accumulation in cancer-initiating cells should be investigated in detail by further analysing the characteristics of DDR inhibition by ΔNp63α in epithelial stem cells.

## Conclusion

Taken together, these results indicate that ΔNp63α represses p53-related radiation-induced DDR and therefore may cause genomic instability in epithelial stem cells after radiation exposure.

## Electronic supplementary material

Below is the link to the electronic supplementary material.

Supplementary Material 1.
**Table S1.** List of primer and siRNA sequences.
**Table S2.** List of primary and secondary antibodies.
Figs. S1-S7.

## Data Availability

RNA-seq files have been uploaded to the DDBJ Sequenced Read Archive under the accession numbers DRR330582-DRR330585.

## List of abbreviations

DDR: DNA damage response
HMEC: human mammary epithelial cell
hiPSC: human induced pluripotent stem cell
iPS-DN: hiPSC-DeltaNp63alpha
iPS-KC: hiPSC-derived keratinocyte
p63-siRNA: sip63
scr: scramble siRNA
