# Supplementary material for: ΔNp63α transcriptionally represses p53 target genes involved in the radiation-induced DNA damage response: ΔNp63α may cause genomic instability in epithelial stem cells
Source: Radiat Oncol. 2022 Nov 15;17:183. doi: 10.1186/s13014-022-02139-7 (PMC9667649; doi:10.1186/s13014-022-02139-7)
Supplement: Supplementary file 1 — Supplementary Material 1. Table S1. List of primer and siRNA sequences. Table S2. List of primary and secondary antibodies. Figs. S1-S7. [file 13014_2022_2139_MOESM1_ESM.docx]

**SUPPLEMENTAL TABLE**

**Table S1.** List of primer and siRNA sequences.

| Gene | Forward 5'->3' | Reverse 5'->3' | Applications |
| --- | --- | --- | --- |
| p63 | GTCACGGAGGTGGTGAAGC | ACATACTGGGCATGGCTGTT | DNA-binding domain |
| ΔNp63 (ΔN) | GGAAAACAATGCCCAGACTC | GGACTGGTGGACGAGGAG | ΔNp63 transactivation domain |
| p63α | CACAGATTGCAGCATTGTCA | ATCGCATGTCGAAATTGCTC | SAM domain |
| TAp63 (TA) | AGTCCAGAGGTTTTCCAGCA | TTGTTTGTCGCACCATCTTC | TAp63 transactivation domain |
| p53 | GTCTGGGCTTCTTGCATTCT | CTCCGTCATGTGCTGTGACT | DNA damage response |
| BAX | CTGAGCAGATCATGAAGACAGG | ATCCTCTGCAGCTCCATGTT |  |
| CDKN1A | CAGCAGAGGAAGACCATGTG | GGCGTTTGGAGTGGTAGAAA |  |
| CDKN2A | CGGAAGGTCCCTCAGACATC | CCCTGTAGGACCTTCGGTGA |  |
| GADD45A | ACGAGGACGACGACAGAGAT | GCAGGATCCTTCCATTGAGA |  |
| NOXA | AAGAAGGCGCGCAAGAAC | TCCTGAGCAGAAGAGTTTGGA |  |
| CK14 | GGCCTGCTGAGATCAAAGAC | CATACTTGGTGCGGAAGTCA | p63 upregulation marker |
| Fst | TCTGCCAGTTCATGGAGGAC | CCCGTTGAAAATCATCCACT |  |
| GPX-2 | TGATTGAGAATGTGGCTTCG | TTTTTGGACAAGGGTGAAGG |  |
| CYGB | AGAAAGTGCCAGGCGAGAT | TCCTCCATGTGCTTGAACTG |  |
| Oct3/4 | GACAGGGGGAGGGGAGGAGCTAGG | CTTCCCTCCAACCAGTTGCCCCAAAC | Yamanaka factor |
| Sox2 | GGGAAATGGGAGGGGTGCAAAAGAGG | TTGCGTGAGTGTGGATGGGATTGGTG |  |
| c-Myc | GCGTCCTGGGAAGGGAGATCCGGAGC | TTGAGGGGCATCGTCGCGGGAGGCTG |  |
| Klf4 | CCCACACAGGTGAGAAACCT | ATGTGTAAGGCGAGGTGGTC |  |
| CD49f | ACGTGATCCGGAAATATGGA | CTCCCGTTCTGTTGGCTCT | Keratinocyto marker |
| CD71 | AAGGCCAATGTCACAAAACC | AAGTCCTCTCCTGGCTCCTC |  |
| BIRC5 | GGACCACCGCATCTCTACAT | TCTCCGCAGTTTCCTCAAAT |  |
| Ki67 | CGTCCCAGTGGAAGAGTTGT | ATATTGCCTCCTGCTCATGG |  |
| GAPDH-1 | GGTGAAGGTCGGAGTCAACG | AATTTGCCATGGGTGGAATC | Internal standard |
| GAPDH-2 | CGAGATCCCTCCAAAATCAA | TTCACACCCATGACGAACAT |  |
|  |  |  |  |
| siRNA sequence |  |  |  |
| siRNA ΔNp63 | GUGUGCUGGUACCUUAUGA-dTdT | UCAUAAGGUACCAGCACAC-dTdT | Target DNA-binding domain |
|  |  |  |  |
| ChIP-qPCR primer |  |  |  |
| Bax promoter | TAAAAATTAACCAGGGGCGG | TCACTGTGTTGCCCAGGCTG |  |
| CDKN1A promoter | CTGTGGCTCTGATTGGCTTT | CCCTTCCTCACCTGAAAACA |  |

**Table S2.** List of primary and secondary antibodies.

| Target antigen | Clone | Supplier | Conjugation | Catalog no. | RRID no. | Species | Experiment | Conc. |
| --- | --- | --- | --- | --- | --- | --- | --- | --- |
| E-cadherin | polyclonal | abcam | unconjugated | Ab53033 | AB_868611 | Rb | IF | 1:200 |
| CK5 | polyclonal | abcam | unconjugated | ab53121 | AB_869889 | Rb | IF | 1:200 |
| CD49f | EPR18124 | abcam | unconjugated | ab181551 | - | Rb | IF | 1:200 |
| p300 | EPR23495-268 | abcam | unconjugated | ab275378 | - | Rb | IF | 1:100 |
| Acetyl-p53 | Lys382 | Cell Signaling Technology | unconjugated | 2525 | AB_330083 | Rb | Wes | 1:1000 |
| p53 | 7F5 | Cell Signaling Technology | unconjugated | 2527 | AB_10695803 | Rb | Wes, ChIP | 1:1000, 1:200 |
| Bax | polyclonal | Cell Signaling Technology | unconjugated | 2772 | AB_10695870 | Rb | Wes | 1:1000 |
| p21 | 12D1 | Cell Signaling Technology | unconjugated | 2947 | AB_823586 | Rb | Wes, IF | 1:1000, 1:50 |
| Cleaved Caspase-3 | Asp175 | Cell Signaling Technology | unconjugated | 9661 | AB_2341188 | Rb | FCM | 1:800 |
| ΔNp63 | E6Q3O | Cell Signaling Technology | unconjugated | 67825 | AB_2799737 | Rb | Wes, IF | 1:1000, 1:1600 |
| GPX2 | polyclonal | Novus biological | unconjugated | NBP3-03209 | - | Rb | Wes | 1:1000 |
| SSEA4 | MC813 | abcam | unconjugated | ab16287 | AB_778073 | Ms | IF | 1:200 |
| ΔNp63(p40) | BC28 | abcam | unconjugated | ab172731 | AB_2891015 | Ms | IF | 1:50 |
| CK14 | LL002 | invitrogen | unconjugated | MA5-11599 | AB_10982092 | Ms | Wes, IF | 1:200, 1:100 |
| γH2AX | JBW301 | Merck | unconjugated | 05-636 | AB_309864 | Ms | IF | 1:800 |
| p63 | 4A4 | Millipore Sigma | unconjugated | MAB4135 | AB_2240873 | Ms | IF | 1:200 |
| GPX2 | 496010 | R&D systems | unconjugated | MAB5470 | AB_2112256 | Ms | IF | 1:100 |
| CYGB | 4C7E11 | proteintech | unconjugated | 60228-1-lg | AB_11182383 | Ms | Wes, IF | 1:500, 1:200 |
| GAPDH | 1E6D9 | proteintech | HRP | HRP-60004 | AB_2737588 | Ms | Wes | 1:4000 |
| p53 | DO-1 | Santa Cruz Biotechnology | unconjugated | sc-126 | AB_628082 | Ms | Wes | 1:300 |
|  |  |  |  |  |  |  |  |  |
| Secondary antibodies |  |  |  |  |  |  |  |  |
| Target antigen | Clone | Supplier | Conjugation | Catalog no. | RRID no. | Species | Experiment | Conc. |
| IgG |  | abcam | AF488 | ab150077 | AB_2630356 | Rb | FCM | 1:1000-2000 |
| IgG |  | abcam | AF594 | ab150076 | AB_2782993 | Rb | IF | 1:200 |
| IgG |  | ThermoFisher Scientific | AF488 | A11029 | AB_2534088 | Ms | IF | 1:200 |
| IgG |  | SantaCruz Biotechnology | HRP | sc-2357 | AB_628497 | Rb | Wes | 1:3000-5000 |
| IgG |  | SantaCruz Biotechnology | HRP | sc-516102 | AB_2687626 | Ms | Wes | 1:3000-5000 |
| Western blot: Wes, Flow Cytometry : FCM, Immunofluorescence: IF, Ms: Mouse, Rb: Rabbit | | | | |  |  |  |  |

**SUPPLEMENTAL FIGURES**


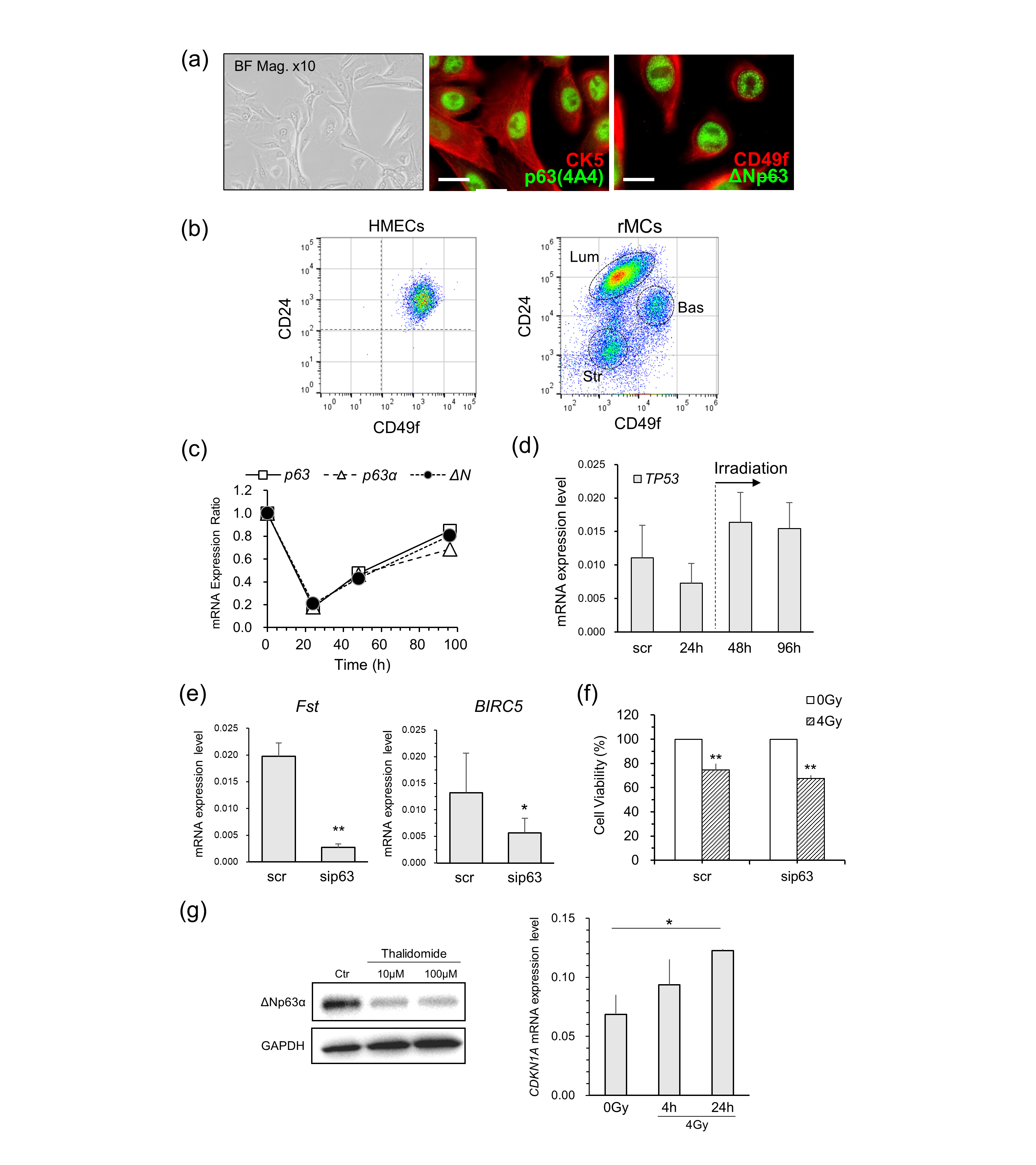


Fig.S1 (a) BF and IF images of HMECs. Anti-p63 (4A4) antibody detects all isoforms of p63. (b) FCM data of HMECs and primary rat mammary gland cells (rMCs), stained by CD49f and CD24 anitibodies, which are major markers of mammary epithelial cells. Lum, Bas, and Str described in figure represent luminal, basal, and stromal cells, respectively. (c) mRNA expression ratios of *p63*, *p63α*, and *ΔN,* shown in Fig.1a. Data were acquired by RT-qPCR in 0-96 h after siRNA treatment, which were normalized with scr-treated HMECs. (d) Time-dependent variation of *TP53* mRNA level of HMECs during 96 h after siRNA treatment. (e) mRNA expression levels of *Fst*, *Birc5*, and *Ki67* after siRNA treatment. Data are means and SE of at least three independent assays. (f) Cell viabilities of HMECs at 48 h post-irradiation, assessed by using 3-(4,5-dimethylthiazol-2-yl)-5-(3-carboxymethoxyphenyl)-2-(4-sulfophenyl)-2H-tetrazolium salt solution (MTS, Promega Inc., Madison, WI). (g) Left panel: Western blotting analysis of ΔNp63α protein expressing in HMECs at 24 h after thalidomide treatment. GAPDH was used as a loading control. Right panel: *CDKN1A* mRNA expression measured by RT-qPCR. All values in mRNA expression data were scaled to the expression level of *GAPDH* as an internal control. *P < 0.05, **P < 0.01 by Student’s *t* test.


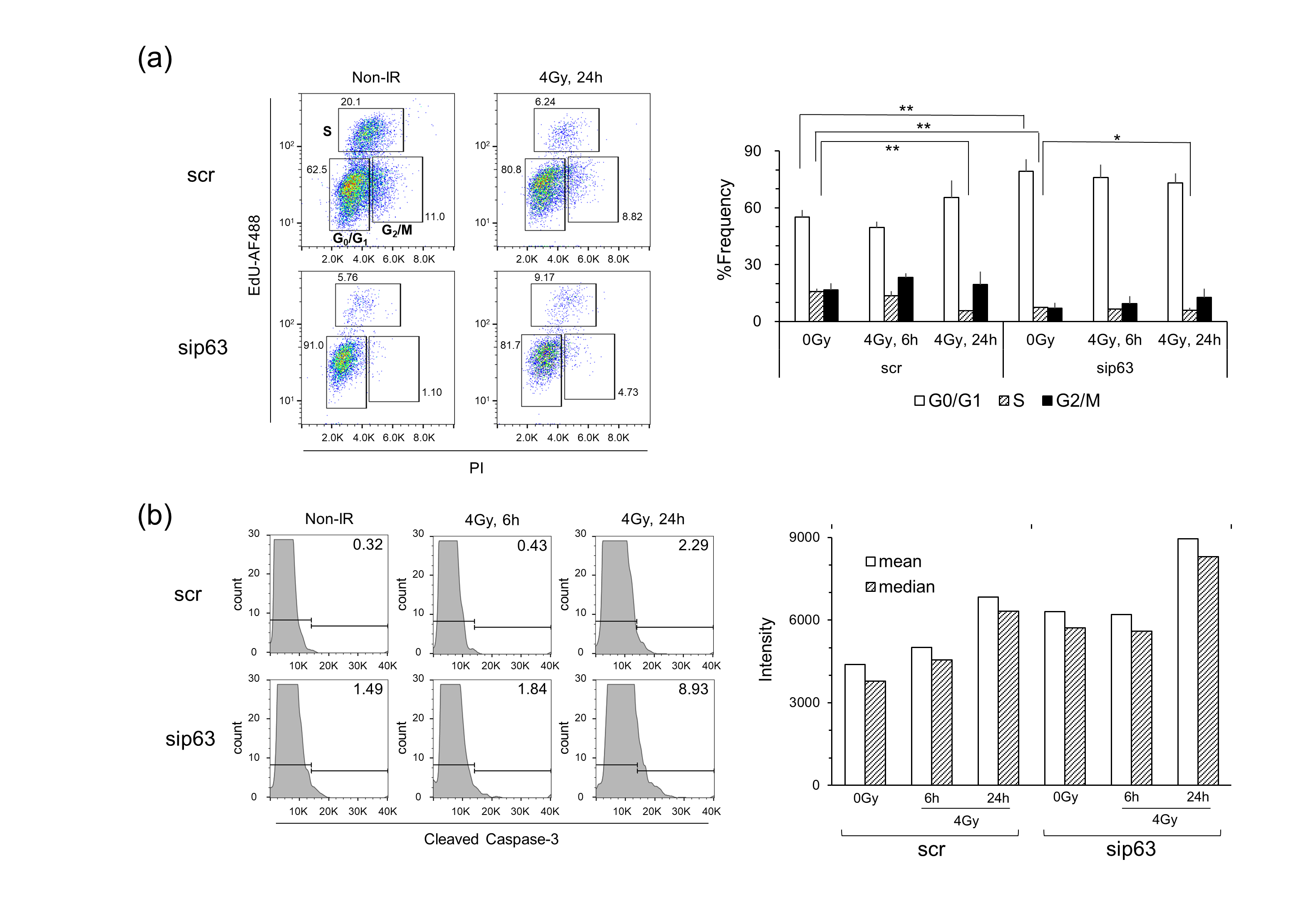


Fig.S2 (a) Representative cell cycle analysis of sip63-treated HMECs after X-irradiation. The left panel shows EdU versus Propidium Iodide (PI) staining plots, where each number in figures represents the frequency of G_0_/G_1_, S, and G_2_/M phase, respectively. The right panel shows the histogram for each phase. Data represent the means and SEs of at least three independent assays. *P < 0.05, **P < 0.01 by Student’s *t* test. (b) The frequencies of CC3-positive apoptotic cells detected by FCM, and mean and median intensities.


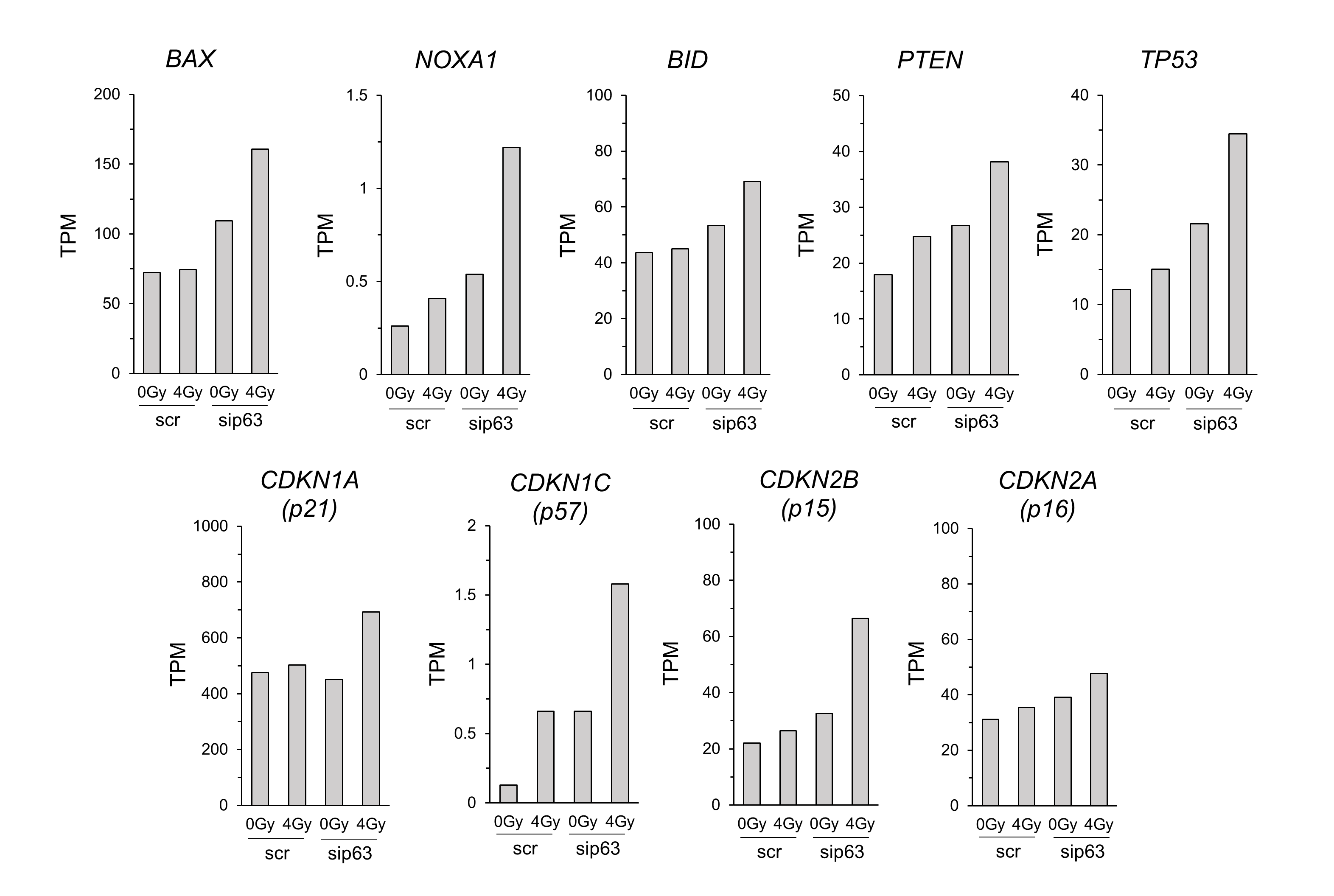


Fig.S3 Normalized expression levels of 10 genes shown as TPM (transcripts per million) from RNA-seq data, partly containing genes corresponding to Fig.1g.


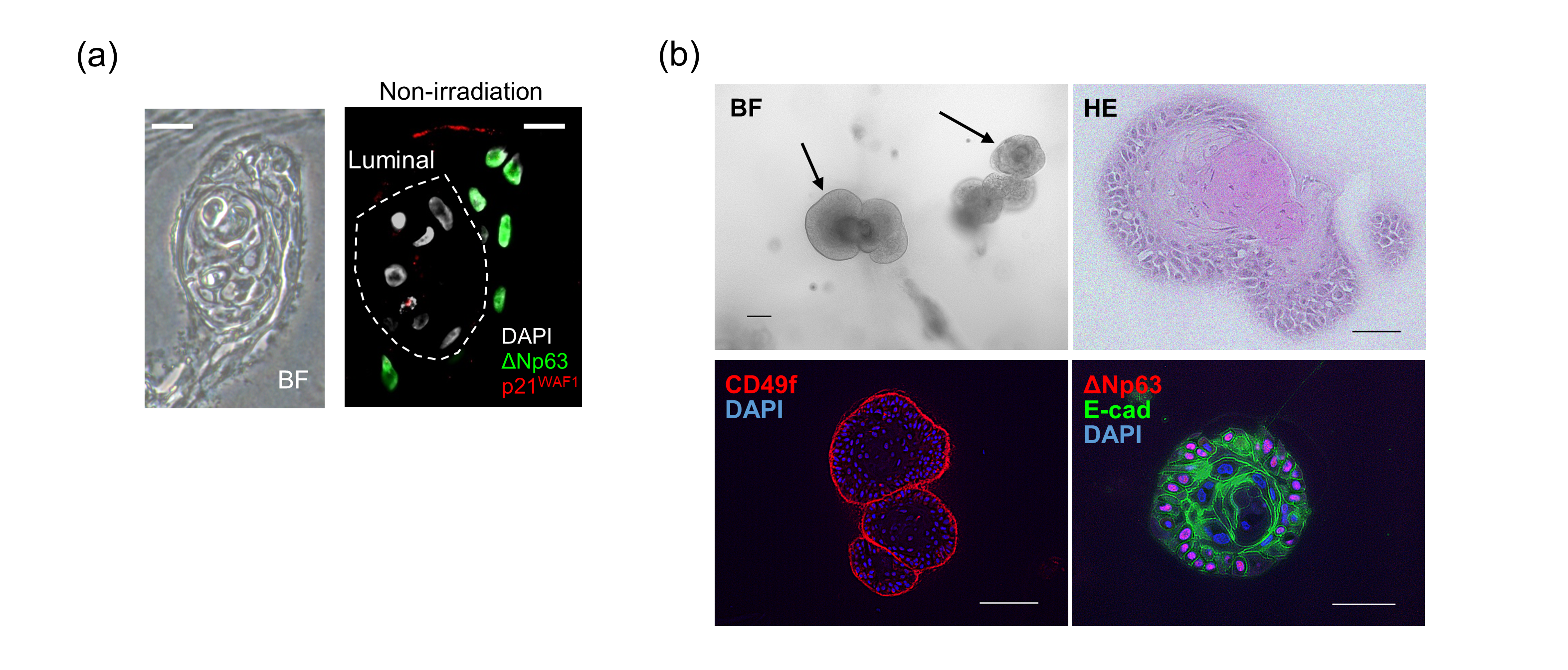


Fig.S4 (a) BF and IF images of mammary organoid generated from single HMEC. IF image shows non-irradiated condition. White, green, and red colors show DAPI, ΔNp63, and p21, respectively. Scale bar, 10μm. (b) BF and IF images of sphere-type colonies generated in collagen gels from single HMEC. E-cad shows anti-E-cadherin antibody. Scale bar, 100μm.


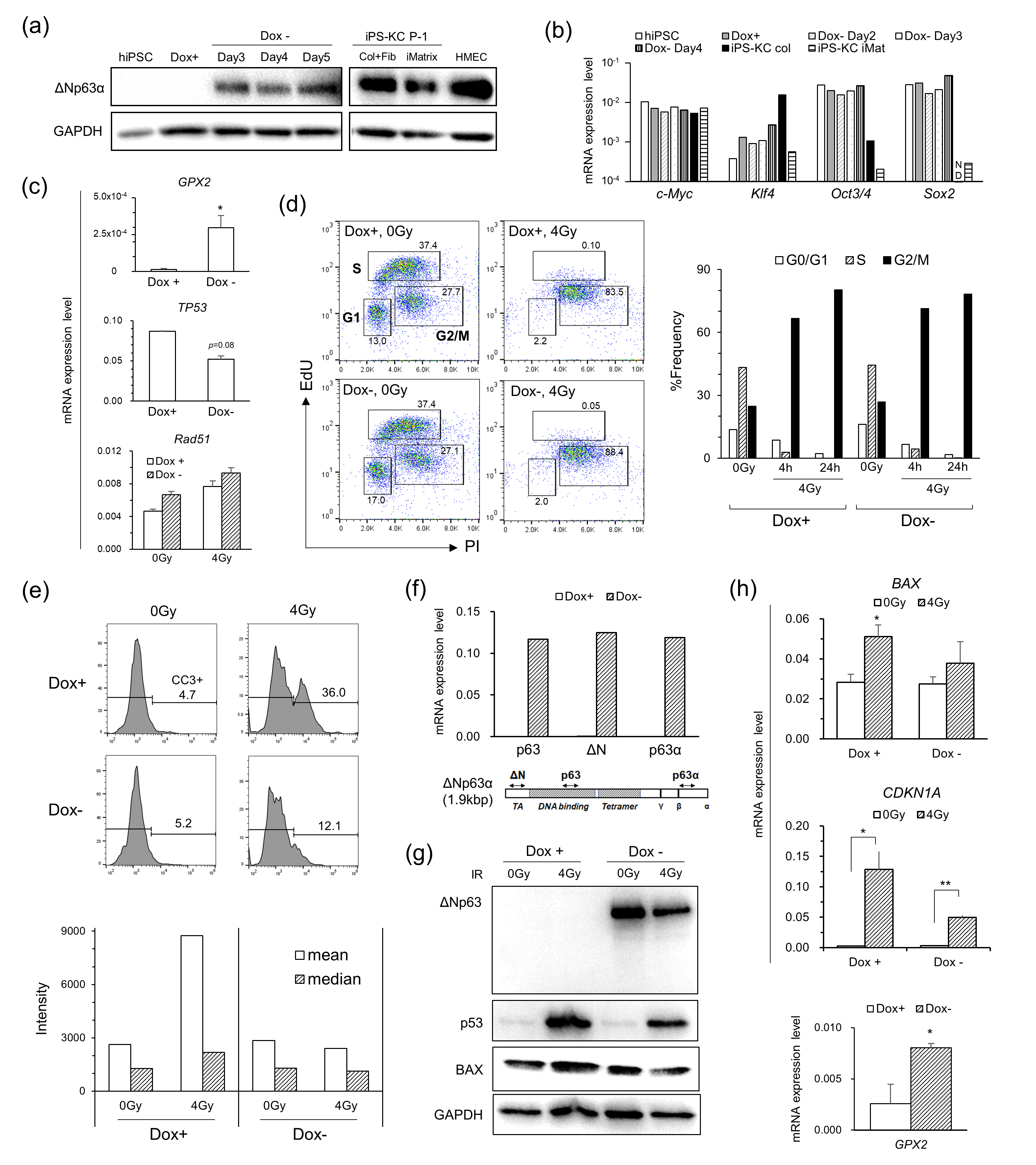


Fig.S5 (a) Detection of ΔNp63α protein expressing in iPS-DN and iPS-KC. GAPDH was used as a loading control. P-1: Passage 1, Col: collagen I coating, Fib: fibronectin coating. (b) iPS cell markers measured by RT-qPCR. All values were scaled to the expression level of *GAPDH* as an internal control. iMat: iMatrix-511 coating. (c) mRNA expression levels of *GPX2*, *TP53*, and *Rad51* in iPS-DN with or without ΔNp63α expression, respectively. (d) Representative cell cycle phases in iPS-DN with or without ΔNp63α expression at 24 h after X-irradiation, which were detected by FCM. (e) Representative FCM data of iPS-DNs post-irradiation, showing the frequency of CC3-positive apoptotic cells (top panel) and the mean and median intensities (bottom panel). (f-h) Analytic results of human B cell-derived iPSCs ectopically expressing ΔNp63α. All data in figure were tested by Student’s *t* test (*P < 0.05, **P < 0.01).


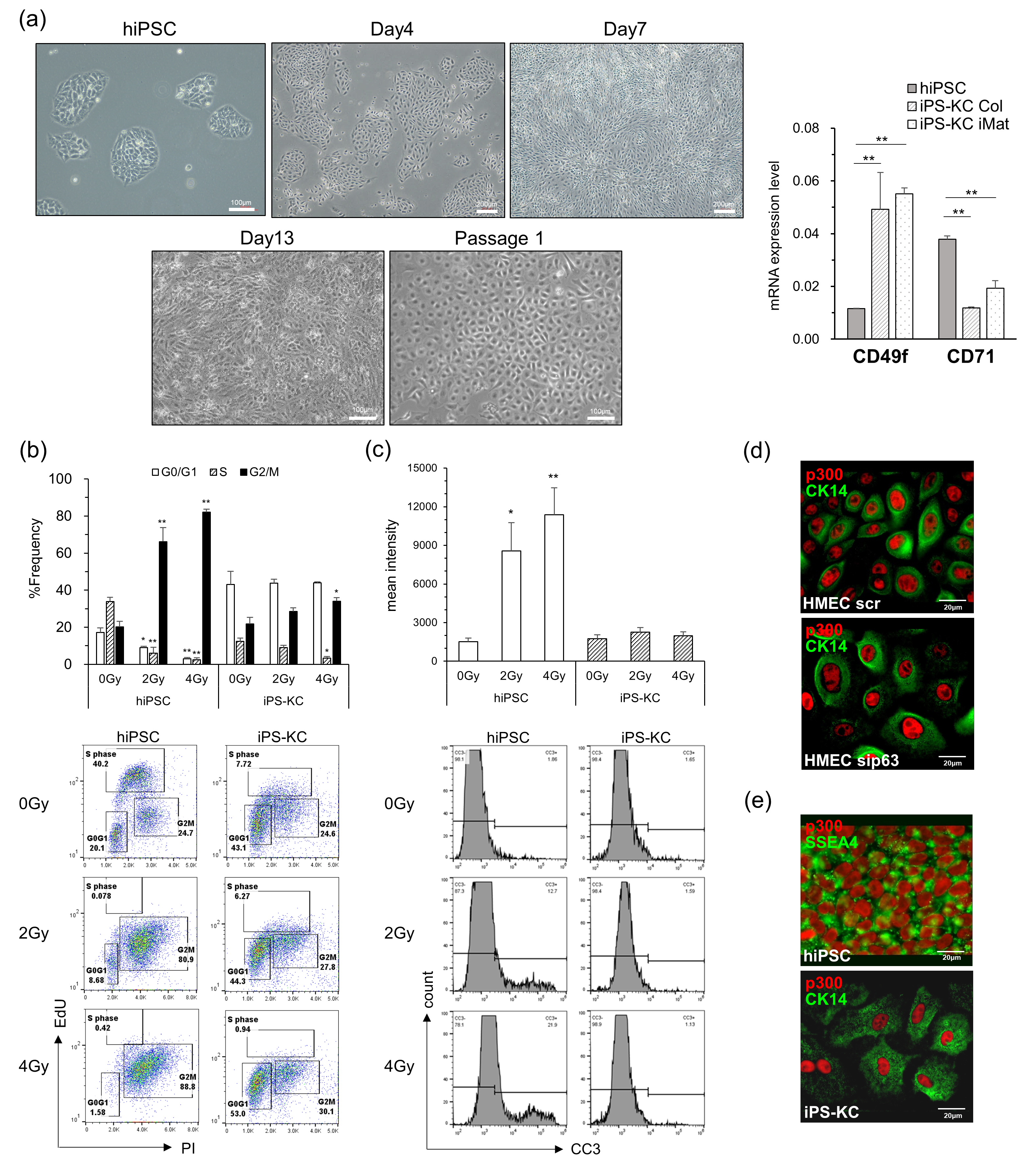


Fig.S6 (a) Left panel: Representative BF images of hiPSCs to iPS-KCs. The treatment of retinoic acid and bone morphogenetic protein 4 was done up to Day 4. Right panel: mRNA expression levels of CD49f and CD71 in hiPSCs and iPS-KCs Passage 1, measured by RT-qPCR. *GAPDH* was used as an internal control. (b) Top panel: the frequencies of each cell cycle phase in hiPSCs and iPS-KCs at 24 h after X-irradiation. Bottom panel: representative EdU/PI plot. (c) Top panel: the mean intensities of hiPSCs and iPS-KCs at 24 h post-irradiation. Bottom panel: representative FCM data post-irradiation. Data represent the means and SEs of at least three independent assays. *P < 0.05, **P < 0.01 by Student’s *t* test. (d,e) IF images of p300 protein expression in HMECs, hiPSCs, and iPS-KCs. CK14 and SSEA-4 were used as a cell marker.


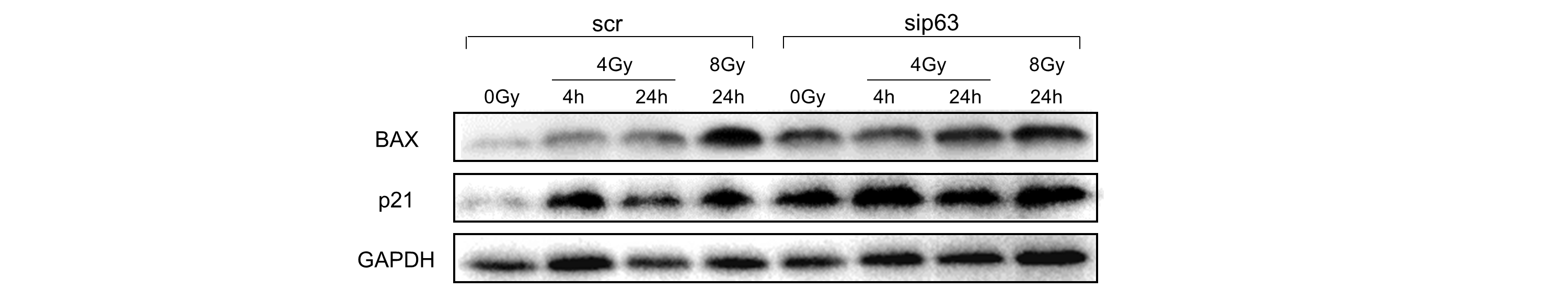


Fig.S7 Overall view of protein assay shown in Fig. 1h. siRNA-treated HMECs were irradiated with 4 or 8 Gy of X-rays, and the proteins were extracted in bulk after a certain time, and then electrophoresed.
